# Pyrrole alkaloids from the fruiting bodies of edible mushroom *Lentinula edodes*†

**DOI:** 10.1039/d3ra02672h

**Published:** 2023-06-16

**Authors:** Zhen-Zhu Zhao, Fei Zhang, Bao-Yu Ji, Ning Zhou, Hui Chen, Yan-Jun Sun, Wei-Sheng Feng, Xiao-Ke Zheng

**Affiliations:** a School of Pharmacy, Henan University of Chinese Medicine Zhengzhou 450046 China fwsh@hactcm.edu.cn zhengxk.2006@163.com

## Abstract

Nine pyrrole alkaloid derivatives, including four new ones (1–4), were isolated from the wild mushroom *Lentinula edodes* for the first time. Their chemical structures were determined using UV-Vis spectroscopy, IR spectroscopy, MS, NMR spectroscopy, and single-crystal X-ray diffraction techniques. Compound 1, a previously unreported bicylo-pyrrole aldehyde homologue, was found to be a major component, approximately 8.2 μg g ^−1^ in the dry powder of *L. edodes*. Compound 1 showed cytotoxicity against SMMC-772 (IC_50_ 15.8 μM) without any cytotoxic effect on LO2, a normal hepatic cell line; compounds 1 and 2 displayed weak immunosuppressive activities by inhibiting the proliferation of induced T cells; compound 3 showed inhibition activity on the proliferation of HaCaT cell line (IC_50_ 25.4 μM) and weak antioxidant activity at a concentration of 50 μM.

## Abstract 2

Nine pyrrole alkaloids were isolated from the wild mushroom *Lentinula edodes* for the first time. Their structures were determined by multiple methods. The novel compound 1 exhibited cytotoxicity against SMMC-772 without any cytotoxic effect on LO2.

## Graphical abstract

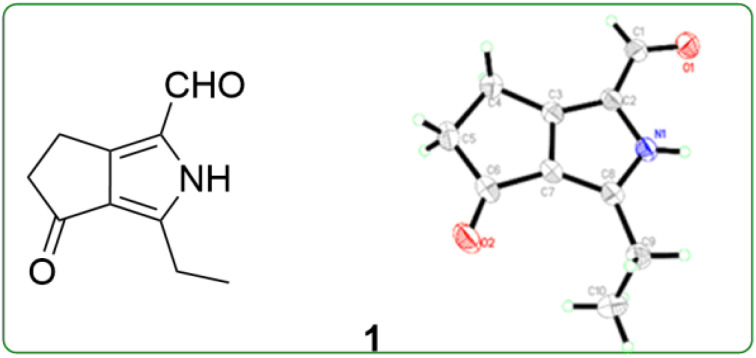

## Introduction

Edible mushrooms are a well-known healthy food for their unique nutritional properties, which have low calories but are rich in nutrients, such as vitamins and minerals. The mushroom *Lentinula edodes*, commonly known as shiitake, is cultivated for both its culinary and medicinal qualities, which has long been utilized as an edible mushroom in East Asia, and its uses were first documented in an ancient Chinese book from the second century, titled Shen Nong Ben Cao Jing (The Divine Farmer's Materia Medica). Lentinan, a high molecular weight neutral polysaccharide purified from a hot water extract of *L. edodes* in 1969,^1^ has been approved as an anti-tumor drug. Subsequently, a series of research was carried out to illustrate the mechanism of lentinan and more active polysaccharides and proteins were found.^2–5^ However, unlike many progresses on biomacromolecule, only a few studies have been conducted to clarify the secondary metabolites (SMs) of the fruiting bodies of *L. edode*s, resulting in the isolation of some active compounds, such as eritadenine and polyacetylene molecules.^6^ Therefore, to complete the possible utilization of *L. edode*s from the perspective of SMs, we studied SMs in this mushroom leading to the isolation of nine pyrrole alkaloids.

Alkaloids are a large group consisting of diverse subgroups of natural products that are most extensively studied in plants. Pyrrole is of special interest in developing new bioactive drugs owing to their anticancer, antimicrobial, antiviral, antimalarial, antitubercular, anti-inflammatory, and enzyme-inhibiting properties,^7–9^*e.g.*, the biggest-selling drug of all time, atorvastatin, a HMG-CoA reductase inhibitor, is just a pyrrole. Indeed, before 2002, diverse pyrrole alkaloids were known mostly from vegetal or marine origin. In mushrooms, the discovery of sciodole from *Tricholoma sciodes*,^10^ an alkaloid containing both pyrrole and indole moieties in its structure, led to the beginning of isolating pyrroles from mushrooms. Subsequently, a series of pyrroles were identified from different mushrooms, however, none of them were from *L. edode*s.^8,11^

**Fig. 1:**
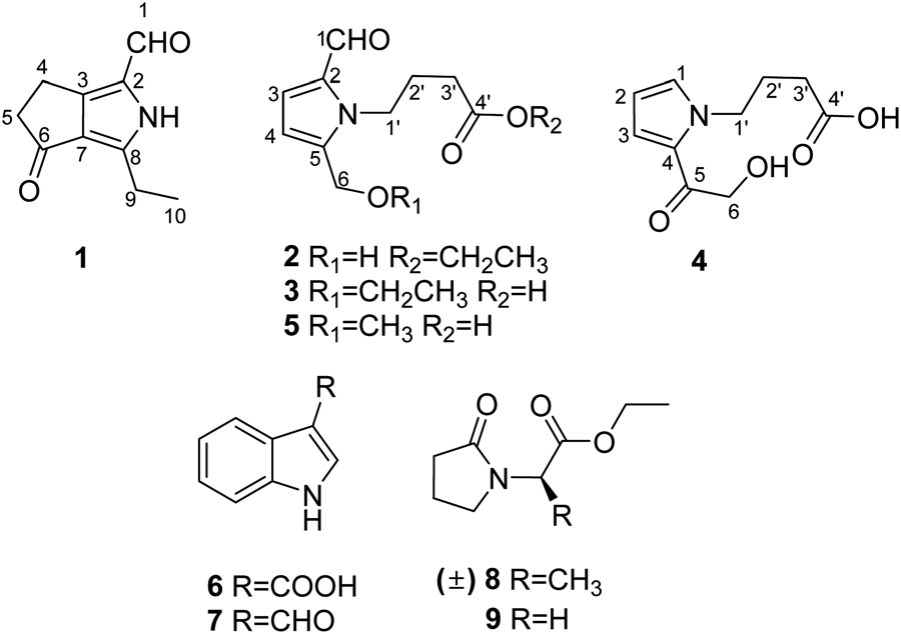
Structures of compounds 1–9.

Herein, we report the isolation and structural elucidation of compounds 1–9 (Fig. 1). Moreover, the potential activities of the isolated compounds 1–5 were evaluated, including inhibitory effects against five human cancer cell lines, a keratinocyte cell line, and T/B cells, as well as free radical scavenging activity on 2,2-diphenyl-1-picrylhydrazyl (DPPH).

## Results and discussion

**Fig. 2:**
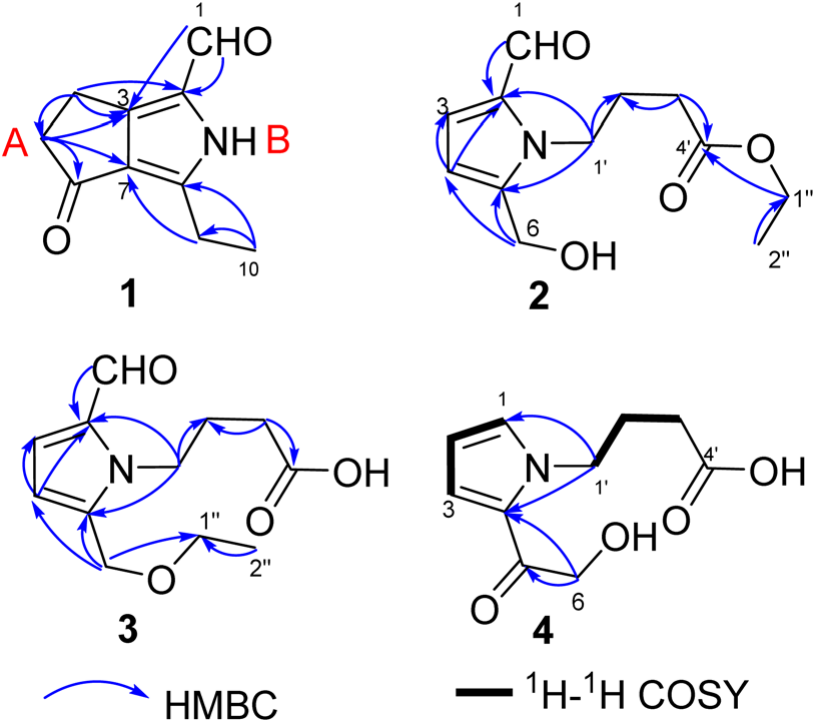
Key 2D NMR correlations of compounds 1–4.

**Fig. 3:**
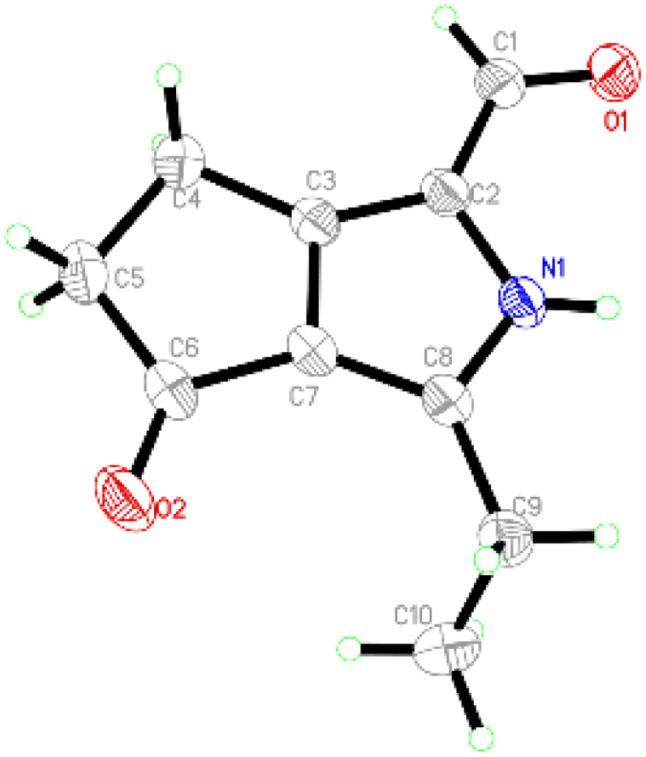
ORTEP drawing of 1.

Compound 1 was obtained as colourless crystals. The molecular formula C_10_H_11_NO_2_ with six sites of unsaturation was established by HRESIMS ion at *m*/*z* 200.0680 [M + Na]^+^ (calcd for C_10_H_11_NO_2_Na, 200.0687). Purified compound 1 showed UV absorption with two absorption maximums at 240 nm and 295 nm. The IR spectrum of 1 revealed the presence of amino (3212 cm^−1^) and two UV-conjugated carbonyl (1691 and 1651 cm^−1^) functionalities. The ^1^H NMR spectrum (Table 1) just revealed 10 protons clearly corresponding to an aldehyde hydrogen [*δ*_H_ 9.46 (s)], a methyl [*δ*_H_ 1.30 (t, *J* = 7.6 Hz)], and three methylenes [*δ*_H_ 2.80 (q, *J* = 7.6 Hz); 2.88 (m); 3.14 (m)]. The ^13^C NMR and DEPT spectra (Table 1) resolved 10 carbon resonances that were ascribed to a methyl (*δ*_C_ 13.4), three methylenes (*δ*_C_ 20.6, 21.4, 43.0), a conjugated aldehyde (*δ*_C_ 178.4), one pair of tetrasubstituted double bounds (*δ*_C_ 125.8, 127.5, 143.5, 155.3), and a conjugated ketone (*δ*_C_ 201.5). After the structure assignment using the 1D NMR data, compound 1 was deduced to possess a bicyclic system for the remaining two degrees of unsaturation. All proton resonances were assigned to their respective carbons *via* the HSQC spectrum. The planner structure of compound 1 comprising two rings, A and B, was successfully established as follows (Fig. 2). The HMBC correlations from H-1 to C-2, from H_3_-10 to C-8/C-9, and from H_2_-9 to C-7 confirmed the presence of the B ring, which constructed a motif of 3-ethyl-pyrrole-1-carbaldehyde. In addition, A ring, a cyclopentenone, was also constructed using the HMBC correlations from H_2_-4 to C-2/C-3/C-5, and from H_2_-5 to C-3/C-6/C-7. The fusion of rings A and B was identified at C-3 and C-7 based on the HMBC experiment (Fig. 2). Therefore, the structure of compound 1 was established as 3-ethyl-4-oxo-2,4,5,6-tetrahydrocyclopenta[*c*]pyrrole-1-carbaldehyde, as shown in Fig. 1, which was further proved by X-ray single crystal experiments (Fig. 3). A comprehensive literature research showed that the architecture of 1 is simple but quite novel.

**Table 1:** ^13^C (150 MHz) and ^1^H (600 MHz) NMR data of compound 1 (*δ* in ppm, CD_3_OD)

No.	*δ*_C_	*δ*_H_ (*J* in Hz)
1	178.4, CH	9.46, s
2	125.8, C	
3	155.3, C	
4	20.6, CH_2_	3.14, m
5	43.0, CH_2_	2.88, m
6	201.5, C	
7	127.5, C	
8	143.5, C	
9	21.4, CH_2_	2.80, q (7.6)
10	13.4, CH_3_	1.30, t (7.6)

Compound 2 was obtained as a colourless oil with a molecular formula of C_12_H_17_NO_4_, determined by HRESIMS ion at *m*/*z* 262.1046 [M + Na]^+^ (calcd for C_12_H_17_NO_4_Na, 262.1050). The UV absorption spectrum of compound 2 showed an absorption maximum at 295 nm and a shoulder at 260 nm. The 1D NMR spectra indicated the presence of a pyrrole ring by displaying a set of two mutual coupled protons [*δ*_H_ 6.99 (1H, d, *J* = 4.0 Hz), H-3; 6.27 (1H, d, *J* = 4.0 Hz), H-4] and four olefinic carbon signals (*δ*_C_ 133.5, C-2; 126.4, CH-3; 111.5, CH-4; 144.6, C-5) (Table 2).^12^ Additionally, an aldehyde [*δ*_H/C_ 9.42 (1H, s)/180.9, CH-1], a hydroxymethyl group [*δ*_H/C_ 4.64 (2H, s)/56.4, CH_2_-6], and three methylene signals [*δ*_H/C_ 2.04 (2H, tt, *J* = 7.4, 7.4 Hz)/27.5, CH_2_-2′; 2.36 (2H, t, *J* = 7.4 Hz)/32.0, CH_2_-3′; *δ*_H/C_ 4.40 (2H, t, *J* = 7.4 Hz)/45.7, CH_2_-1′] as well as a carbonyl signal (*δ*_C_ 174.7, C-4′) were also observed in the 1D NMR and HSQC spectra. The above NMR data of 2 showed a close resemblance to those of compound 5,^12^ while the appearance of the signals for an ethoxyl group [*δ*_H/C_ 1.25 (3H, t, *J* = 7.1 Hz)/14.5; *δ*_H/C_ 4.12 (2H, q, *J* = 7.1 Hz)/61.7] indicated that compound 2 (Table 2) was an ethylated derivative of 4-[2-formyl-5-hydroxymethyl)-1*H*-pyrrol-1-yl]butanoic acid (5). Then, the HMBC correlations from H_2_-1′′ to C-4′ assigned this ethoxyl group to C-4′ (Fig. 2). Therefore, the structure of 2 was determined as ethyl 4-[2-formyl-5-(hydroxymethyl)-1*H*-pyrrol-1-yl]butanoate (Fig. 1).

**Table 2:** ^13^C (150 MHz) and ^1^H (600 MHz) NMR data of compounds 2–4 (*δ* in ppm, CD_3_OD)

No.	2		3		4	
	*δ*_C_	*δ*_H_ (*J* in Hz)	*δ*_C_	*δ*_H_ (*J* in Hz)	*δ*_C_	*δ*_H_ (*J* in Hz)
1	180.9, CH	9.42, s	181.1, CH	9.44, s	132.9, CH	7.13, dd (2.1,1.2)
2	133.5, C		133.7, C		109.9, CH_2_	6.18, dd (4.1,2.1)
3	126.4, CH	6.99, d (4.0)	126.0, CH	6.99, d (4.0)	120.8, CH	7.09, dd (4.1,1.2)
4	111.5, CH	6.27, d (4.0)	112.7, CH	6.28, d (4.0)	127.9, C	
5	144.6, C		141.4, C		190.1, C	
6	56.4, CH_2_	4.64, s	64.5, CH_2_	4.54, s	65.4, CH_2_	4.68, s
1′	45.7, CH_2_	4.40, t (7.4)	46.0, CH_2_	4.37, t (7.7)	50.0, CH_2_	4.41, t (6.7)
2′	27.5, CH_2_	2.04, tt (7.4, 7.4)	27.7, CH_2_	2.01, tt (7.7, 7.3)	29.0, CH_2_	2.00, m
3′	32.0, CH_2_	2.36, t (7.4)	32.0, CH_2_	2.33, t (7.3)	34.5, CH_2_	2.17, m
4′	174.7, C		176.9, C		Unobserved C	
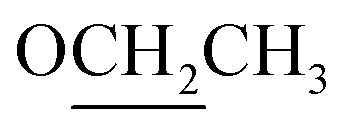	61.7, CH_2_	4.12, q (7.1)	66.9, CH_2_	3.55, q (7.0)		
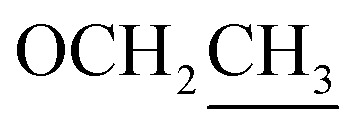	14.5, CH_3_	1.25, t (7.1)	15.4, CH_3_	1.20, t (7.0)		

Compounds 3 and 2 are isomers by showing the same molecular formula, C_12_H_17_NO_4_, while a further comparison of the 1D NMR data between 3 and 2 (Table 2) indicated that compound 3 was also a derivative of 4-[2-formyl-5-(hydroxymethyl)-1*H*-pyrrol-1-yl]butanoic acid. However, compound 3 is an etherified derivative at HOCH_2_-6 for the obvious differences in chemical shifts of the ethoxyl and hydroxymethyl groups in the ^13^C NMR spectra [3: OCH_2_CH_3_ (*δ*_C_ 66.9, 15.4), OCH_2_-6 (*δ*_C_ 64.5); 2: OCH_2_CH_3_ (*δ*_C_ 61.7, 14.5), OCH_2_-6 (*δ*_C_ 56.4)], which was also supported by HMBC correlations from H_2_-6 to C-1′′ (Fig. 2). Finally, the structure of 3 was defined as 4-[2-formyl-5-(ethoxymethyl)-1*H*-pyrrol-1-yl]butanoic acid (Fig. 1).

Compound 4 was isolated as a colourless oil, and its molecular formula was C_10_H_13_NO_4_ deduced by the HRESIMS ion at *m*/*z* 234.0739 [M + Na]^+^ (calcd for C_10_H_13_NO_4_Na, 234.0737). The IR spectrum of 4 revealed the presence of a hydroxyl (3368, cm^−1^) and one carbonyl (1677 and 1645 cm^−1^) functionalities. Similar to the structures of compounds 3 and 5, compound 4 conserved the framework of (1*H*-pyrrol-1-yl)butanoic acid due to the resembling chemical shifts and coupling constants (Table 2). Whereas, instead of the obvious aldehyde signal, the newly appeared signal of pyrrole hydrogen [*δ*_H_ 7.13 (1H, dd, *J* = 2.1, 1.2 Hz)] suggested that CHO-1 was degraded in 4, which was consistent with ^1^H–^1^H COSY and HMBC spectra (Fig. 2). In addition, HMBC correlations from H_2_-6 [*δ*_H_ 4.68 (s)] to C-5 (*δ*_C_ 190.1) and C-4 (*δ*_C_ 127.9) successfully assigned the side chain –COCH_2_OH to C-4. Thus, the structure of 4 is 4-[5-(2-hydroxymethyl-1-carbonyl)-1*H*-pyrrol-1-yl]butanoic acid (Fig. 1).

By analysis and comparison of their NMR data with that documented in the literature, the known compounds 5–9 were identified as 4-[2-formyl-5-(hydroxymethyl)-1*H*-pyrrol-1-yl]butanoic acid (5),^12^ indole-3-acetic acid B (6),^13^ indole-3-carboxaldehyde (7),^14^ ethyl (±)2-(2-oxopyrrolidin-1-yl)propanoate (8),^15^ and ethyl 2-(2-oxopyrrolidin-1-yl)acetate (9)^16^ (Fig. 1), respectively. Since compounds 8 and 9 are uncomman natural products, their 1D NMR spectra are also listed in the ESI.†

As compounds 1–4 are new compounds, and compound 5 is a derivative of 1–4, they were chosen to test for their inhibitory effects against five human cancer cell lines, a keratinocyte cell line, and T/B cells, as well as DPPH-free radical scavenging activity. Among them, compound 1 exhibited inhibitory activity against SMMC-7721 without any cytotoxic effect on normal hepatocyte, LO2 (Table 3); compounds 1 and 2 showed weak anti-proliferation of induced T cells (Table 4); compound 3 displayed activities against proliferation of HaCaT cell line and scavenging of the DPPH free radical (Tables 3 and 5).

**Table 3:** IC_50_ (μM) values of anti-proliferative effects of compound 1 and 3^a^

Samples	SMMC-7721	LO2	HaCaT
1	15.8 ± 0.9	>40	>40
3	>40	—a	25.4 ± 0.3
Cisplatin	12.3 ± 0.3	2.9 ± 0.2	—a
Taxol	<0.008	<0.008	—a
MTX	—a	—a	41.2 ± 0.8

a “—” means that this compound was not tested in the corresponding experiment.

**Table 4:** Inhibition activities against the proliferation of induced T cells by ConA of compounds 1 and 2 (20 μM)

Samples	SI	Inhibition rate (%)
1	2.8 ± 0.1^a^	15.0 ± 2.5
2	2.3 ± 0.3^a^	31.1 ± 7.6
DEX	0.5 ± 0.1^b^	83.6 ± 3.4
M	3.3 ± 0.1^c^	—
Con	1.0 ± 0.3	—

a *P* < 0.05 *vs.* M group.

b *P* < 0.01 *vs.* M group.

c *P* < 0.01 *vs.* Con group.

**Table 5:** DPPH free radical scavenging activity of compound 3

Samples	*C* (μM)	Antioxidant rate (%)
Trolox	25	94.8 ± 0.0
3	50	12.9 ± 2.4

## Conclusions

In summary, four new pyrrole alkaloid derivatives (1–4) and five known ones (5–9) with various bioactivities were isolated from the wild edible mushroom *L. edodes* by alcohol (95%) extraction followed by ethyl acetate fractionation. Compound 1, bearing a novel cyclopenta[*c*]pyrrole-1-carbaldehyde scaffold, could inhibit the proliferation of SMMC-7721 without any cytotoxic effect on normal hepatocytes, showing the value for further research. Undoubtedly, this work not only enriches the ingredients of *L. edodes*, which is beneficial for the public's knowledge of it, but also provides solid evidence for *L. edodes* used as a functional food.

## Experimental section

### General experimental procedures

Optical rotation studies were performed on a JASCO P-1020 digital polarimeter (Horiba, Kyoto, Japan). UV spectra were recorded on a Shimadzu UV-2401PC UV-visible recording spectrophotometer (Shimadzu, Kyoto, Japan). IR spectra were obtained on a Thermo Scientific Nicolet iS10 FT-IR Spectrometer (Thermo, Waltham, MA, USA). 1D and 2D NMR spectra were obtained on a Bruker Avance III 600 MHz spectrometer (Bruker Corporation, Karlsruhe, Germany) instrument. HRESIMS were recorded on an Agilent 6200 Q-TOF MS system (Agilent Technologies, Santa Clara, CA, USA). Single crystal X-ray diffraction was performed on a Bruker D8 Quest (Karlsruhe, Germany). The melting point was measured on an X-4 microscopic melting point meter (Yuhua Instrument Co., Ltd, Gongyi, China). Sephadex LH-20 (Amersham Biosciences, Uppsala, Sweden) and silica gel (Qingdao Haiyang Chemical Co., Ltd) were used for column chromatography (CC). Medium pressure liquid chromatography (MPLC) was performed on a Büchi Sepacore System equipped with a pump manager C-615, a pump modules C-605, a fraction collector C-660 (Büchi Labortechnik AG, Flawil, Switzerland), and a column packed with Chromatorex C-18 (dimensions 450 mm × i.d. 14 mm, particle size: 40–75 μm, Fuji Silysia Chemical Ltd., Kasugai, Japan). Preparative high-performance liquid chromatography (prep. HPLC) was performed on an Agilent 1260 liquid chromatography system equipped with Zorbax SB-C18 columns (particle size 5 μm, dimensions 150 mm × i.d. 9.4 mm or 150 mm × i.d. 21.2 mm, flow rate 7 or 15 mL min^−1^, respectively) and a DAD detector (Agilent Technologies, Santa Clara, CA, USA).

### Fungal material

Fruiting bodies of the wild mushroom *L. edodes* were purchased from Lijiang City, Yunnan Province, China in 2016, and were identified by the molecular evidence of ITS gene fragment (GenBank accession no. OQ680131), whose BLAST search result showed that the sequence was similar (>99%) to the sequence of *L. edodes*. A voucher specimen (no. HZYXG01) was deposited at the School of Pharmacy, Henan University of Chinese Medicine.

### Extraction and isolation

The dried fruiting bodies of *L. edodes* (3.5 kg) were soaked in 20 L EtOH (95%) at room temperature for three days (×4). Then, a total of 80 L solvents were concentrated till about 5 L under reduced pressure, which was further extracted by EtOAc (5 L × 3). The removal of all solvents gave an EtOAc layer (120 g). The crude extract was separated over silica gel flash CC, eluted with a gradient system of petroleum ether-acetone, to produce seven fractions, A to G. Fraction E (24 g) was divided on MPLC, eluted with CH_3_OH–H_2_O (v/v, from 20/80 to 0/100), yielding 14 subfractions, E1–E14. Subsequently, fractions E4 (CH_3_OH), E5 (acetone), and E6 (acetone) were subjected to a Sephadex LH-20 unit each affording 11 (E4a–E4k), 9 (E5a–E5i) and 12 fractions (E6a–E6l).

Compounds 1 (28.7 mg, *t*_R_ = 6.0 min) from fraction E4c, 4 (2.2 mg, *t*_R_ = 9.5 min) from fraction E4d, 6 (1.6 mg, *t*_R_ = 14.1 min) from fraction E4j, and 7 (2.8 mg, *t*_R_ = 14.3 min) from fraction E4j were purified by prep. HPLC (CH_3_CN/H_2_O: 25/75 to 50/50, 25 min, flow rate 15 mL min^−1^). Compounds 2 (5.5 mg, *t*_R_ = 10.0 min, CH_3_CN/H_2_O: 15/85 to 30/70) from fraction E5c, and 3 (2.6 mg, *t*_R_ = 11.8 min, CH_3_CN/H_2_O: 15/85 to 35/65) from fraction E6f were obtained by prep. HPLC (20 min, flow rate 7 mL min^−1^).

Fraction E4a was subjected to a Sephadex LH-20 (acetone) unit affording four minor fractions (E4a1–E4a4). Compounds 5 (3.2 mg, *t*_R_ = 14.6 min) from fraction E4a3, 8 from fraction E4a3 (3.1 mg, *t*_R_ = 8.1 min), and 9 (4.4 mg, *t*_R_ = 5.4 min) from fraction E4a4 were obtained by prep. HPLC (CH_3_CN/H_2_O: 35/65 to 60/40, 25 min, flow rate 15 mL min^−1^).

3-Ethyl-4-oxo-2,4,5,6-tetrahydrocyclopenta[*c*]pyrrole-1-carbaldehyde (1): colourless block crystals; mp 140.2–148.2 °C; UV (MeOH) *λ*_max_ nm (log *ε*): 195 (5.17), 240 (5.04), 295 (5.03); IR (MeOH) *ν*_max_ cm^−1^: 3212,1691, 1651, 1576, 1520, 1466, 1291, 1025, 875; ^1^H NMR (600 MHz CD_3_OD) data see Table 1; ^13^C NMR (150 MHz CD_3_OD) data see Table 1; HRESIMS *m*/*z* 200.0680 [M + Na]^+^ (calcd for C_10_H_11_NO_2_Na, 200.0687), and *m*/*z* 178.0860 [M + H]^+^ (calcd for C_10_H_12_NO_2_, 178.0868).

Single crystal X-ray diffraction data for 1: a block-like specimen of C_10_H_11_NO_2_, approximate dimensions 0.180 mm × 0.330 mm × 0.370 mm, was used for the X-ray crystallographic analysis on the Bruker D8 Quest instrument. The integration of the data using a monoclinic unit cell yielded a total of 14 437 reflections to a maximum *θ* angle of 74.55° (0.80 Å resolution), of which 1838 were independent (average redundancy 7.855, completeness = 98.9%, *R*_int_ = 2.99%, *R*_sig_ = 2.11%) and 1775 (96.57%) were greater than 2*σ*(*F*^2^). The final cell constants of *a* = 6.7030(4) Å, *b* = 9.0452(6) Å, *c* = 15.0090(9) Å, *β* = 93.234(2)°, volume = 908.55(10) Å^3^, *T* = 273(2) K. Data were corrected for absorption effects using the multi-scan method (SADABS). The structure was solved and refined using the Bruker SHELXTL Software Package, using the space group *P*121/*n*1, with *Z* = 4, *μ*(CuKα) = 1.54178. The final anisotropic full-matrix least-squares refinement on *F*^2^ with 119 variables converged at *R*_1_ = 3.97%, for the observed data and w*R*_2_ = 11.07% for all data. The goodness-of-fit was 1.056. These data can be obtained free of charge *via*https://www.ccdc.cam.ac.uk (CCDC no. 2251050).

4-[2-Formyl-5-(hydroxymethyl)-1*H*-pyrrol-1-yl]butanoate (2): a colourless oil; UV (MeOH) *λ*_max_ nm (log *ε*): 195 (4.71), 295 (4.92); IR (MeOH) *ν*_max_ cm^−1^: 3382, 1732, 1659, 1379, 1186, 1032, 783; ^1^H NMR (600 MHz CD_3_OD) data see Table 2; ^13^C NMR (150 MHz CD_3_OD) data see Table 2. HRESIMS *m*/*z* HRESIMS *m*/*z* 262.1046 [M + Na]^+^ (calcd for C_12_H_17_NO_4_Na 262.1050).

4-[2-Formyl-5-(ethoxymethyl)-1*H*-pyrrol-1-yl]butanoic acid (3): a colourless oil; UV (MeOH) *λ*_max_ nm (log *ε*): 195 (4.87), 290 (4.93); IR (MeOH) *ν*_max_ cm^−1^: 3416, 1658, 1645, 1555, 1411, 1175, 1088, 782; ^1^H NMR (600 MHz CD_3_OD) data see Table 2; ^13^C NMR (150 MHz CD_3_OD) data see Table 2; HRESIMS *m*/*z* 262.1055 [M + Na]^+^ (calcd for C_12_H_17_NO_4_Na, 262.1050).

4-[5-(2-Hydroxymethyl-1-carbonyl)-1*H*-pyrrol-1-yl]butanoic acid (4): a colourless oil, UV (MeOH) *λ*_max_ nm (log *ε*): 195 (4.04), 295 (2.86); IR (MeOH) *ν*_max_ cm^−1^: 3368, 1677, 1645, 1570, 1425, 1114, 1087. ^1^H NMR (600 MHz CD_3_OD) data see Table 2; ^13^C NMR (150 MHz CD_3_OD) data see Table 2; HRESIMS *m*/*z* 234.0739 [M + Na]^+^ (calcd for C_10_H_13_NO_4_Na 234.0737).

Ethyl (±)2-(2-oxopyrrolidin-1-yl)propanoate (8): a colourless oil; ^13^C NMR data measured in CD_3_OD *δ*_C_ 14.5 
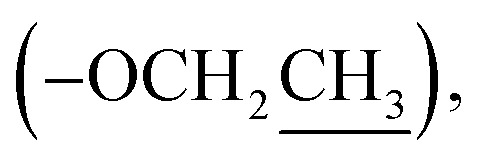
 14.9 (CH_3_-3′), 19.1 (CH_2_-3), 31.9 (CH_2_-2), 45.4 (CH_2_-4), 51.2 (CH-1′), 62.5 
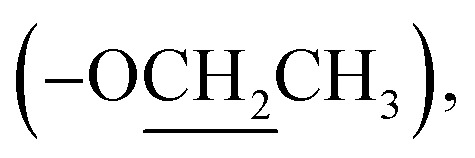
 172.6 (C-2′), 178.0 (C-1).

Ethyl 2-(2-oxopyrrolidin-1-yl)acetate (9): a colourless oil; ^13^C NMR data measured in CD_3_OD *δ*_C_ 14.4 
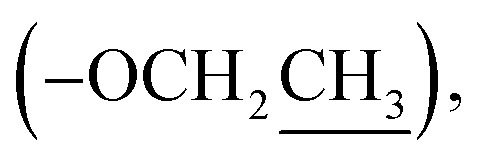
 18.9 (CH_2_-3), 31.4 (CH_2_-2), 45.0 (CH_2_-4), 49.3 (CH-1′), 62.5 
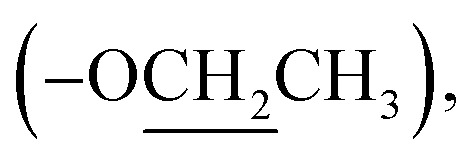
 170.2 (C-2′), 178.5 (C-1).

### Biological assays

#### Cytotoxicity against five human cancer cell lines and a keratinocyte cell line

Five human cancer cell lines were used: HL-60 (ATCC CCL-240) human myeloid leukemia; SMMC-7721 human hepatocellular carcinoma; A-549 (ATCC CCL-185) lung cancer; MCF-7 (ATCC HTB-22) breast cancer; SW-480 (ATCC CCL-228) human colon cancer. The cell line SMMC-7721 was bought from China Infrastructure of Cell Line Resources (Beijing, China), and others were bought from American Type Culture Collection (ATCC, Manassas, VA). The five human cancer cell lines were cultured in RPMI-1640 medium containing 10% fetal bovine serum (FBS) (Hyclone) and maintained at 37 °C under 5% CO_2_ in a humidified atmosphere. The keratinocyte cell line was HaCaT (FH0186) bought from Fufeng Biology (Shanghai, China), and this cell line was cultured in a DMEM medium containing 10% FBS. CCK-8 (Beijing Solarbio Science & Technology Co., Ltd, Beijing, China) was used to assess the cell viability. In brief, each well of a 96-well cell culture plate was seeded with 100 μL of adherent cells (1 ×10^5^ cells per mL) and kept for 12 h for adherence and followed by the addition of the test compounds. However, 100 μL suspended cells (1 × 10^5^ cells per mL) were seeded before being added the test compounds. After adding different concentrations of the test compounds, each cell line was incubated for 48 h. Cisplatin, Taxol, and methotrexate (MTX) were used as positive controls. After the incubation, each well was treated with CCK-8 (10 μL) and incubation was continued for 4 h at 37 °C. Then, the 96-well cell culture plates were subjected to a measure of optical density at 450 nm with a 96-well microplate reader (SpectraMax iD3, Molecular Devices, LLC.). The IC_50_ value for each compound was calculated by the Reed and Muench method. All experiments were performed in triplicate.

#### Immunosuppressive activities assay

The RPMI 1640 medium containing 10% FBS was used to culture the murine spleen cells. CCK-8 was used to assess cell viability. Compounds 1–5 were subjected to evaluate their inhibition on the proliferation of T and B lymphocytes. The fresh spleen cells were from BALB/c mice (7–9 weeks old, Beijing Huafukang Biotechnology Co., Ltd). The cell mixture was dispensed into 96-well plates (2 × 10^6^ cells per mL), in the absence or presence of compounds (*c* = 20.0 μM), and were stimulated with ConA (5 μg mL^−1^) or LPS (15 μg mL^−1^) to induce T cell or B cell proliferative responses, respectively. These 96-well plates were maintained at 37 °C under 5% CO_2_ in a humidified atmosphere for 48 h in triplicate. Dexamethasone (DEX) (*c* = 10.0 μM) was used as a positive control. In the 96-well plates, the wells only filled with cells and ConA/LPS were assigned as the modal group (M), the wells just containing cells were described as the control group (Con), and the wells only containing culture medium were described as the blank group. 10 μL of CCK-8 was added to each well at the final 4–5 h of culture, and then the absorbance (OD) values were measured with a microplate reader at 450 nm. Stimulation Index (SI) = (OD_sample_ − OD_blank_)/(OD_Con_ − OD_blank_); inhibition rate = (1 − SI_sample_/SI_M_) × %.

#### The DPPH free radical scavenging assay

This assay was modified slightly according to the previously reported method.^17^ Compounds 1–5 (at a final concentration of 50 μM) were mixed with DPPH (at a final concentration of 100 μM) in ethanol. After incubation in the dark at 30 °C for 1 h, the OD values were measured at 515 nm. Trolox (at a final concentration of 25 μM) was used as the reference antioxidant. In the 96-well plates, the wells just containing DPPH were described as the control group (Con), and the wells only containing solvent were assigned as the blank group. The assay was carried out in triplicate. Antioxidant rate = [1 − (OD_sample_ − OD_blank_)/(OD_Con_ − OD_blank_)] × %.

#### Statistical analysis method

SPSS 22.0 software was used for the statistical analysis of the data, which was expressed as *X̄* ± *s*. One-way ANOVA was used to compare the differences between groups. LSD or Dunnett's T3 test was used for pair comparison according to standard deviation values. *P* < 0.05 or *P* < 0.01 were considered statistically significant.

## Conflicts of interest

There are no conflicts to declare.

## Supplementary Material

RA-013-D3RA02672H-s001

RA-013-D3RA02672H-s002
