# Supplementary material for: Pyrrole alkaloids from the fruiting bodies of edible mushroom Lentinula edodes
Source: RSC Adv. 2023 Jun 16;13(27):18223–8. doi: 10.1039/d3ra02672h (PMC10273186; doi:10.1039/d3ra02672h)

Electronic Supplementary Material (ESI) for RSC Advances.
This journal is © The Royal Society of Chemistry 2023

Pyrrole Alkaloids from the Fruiting Bodies of the Edible Mushroom *Lentinula edodes*

Zhen-Zhu Zhao, Fei Zhang, Bao-Yu Ji, Ning Zhou, Hui Chen, Yan-Jun Sun, Wei-Sheng Feng*, Xiao-Ke Zheng*

School of Pharmacy, Henan University of Chinese Medicine, Zhengzhou 450046, China

Corresponding Authors

fwsh@hactcm.edu.cn (W.-S. Feng); zhengxk.2006@163.com (X. -K. Zheng)

Contents

1. Spectroscopic data......3
Figure S1. 1H NMR spectrum of **1** (600 MHz, CD_3OD)......3
Figure S2. ^{13}C and DEPT NMR spectra of **1** (150 MHz, CD_3OD)......3
Figure S3. HMBC spectrum of **1** 4
Figure S4. HRESIMS report of **1**......5
Figure S5. IR report of **1** 6
Figure S6. ^1H NMR spectrum of **2** (600 MHz, CD_3OD) 7
Figure S7. ^{13}C and DEPT NMR spectra of **2** (150 MHz, CD_3OD)......7
Figure S8. HSQC spectrum of **2** 8
Figure S9. HMBC spectrum of **2** 8
Figure S10. HRESIMS report of **2**......9
Figure S11. IR report of **2** 10
Figure S12. ^1H NMR spectrum of **3** (600 MHz, CD_3OD) 11
Figure S13. ^{13}C and DEPT NMR spectra of **3** (150 MHz, CD_3OD)......11
Figure S14. HSQC spectrum of **3** 12
Figure S15. HMBC spectrum of **3** 12
Figure S16. HRESIMS report of **3**......13
Figure S17. IR report of **3** 14
Figure S18. ^1H NMR spectrum of **4** (600 MHz, CD_3OD) 15
Figure S19. ^{13}C and DEPT NMR spectra of **4** (150 MHz, CD_3OD)......15
Figure S20. HSQC spectrum of **4** 16
Figure S21. ^1H-^1H COSY spectrum of **4**......16
Figure S22. HMBC spectrum of **4** 17
Figure S23. HRESIMS report of **4**......18
Figure S24. IR report of **4** 19
Figure S25. ^1H NMR spectrum of **5** (600 MHz, CD_3OD) 20
Figure S26. ^{13}C and DEPT NMR spectra of **5** (150 MHz, CD_3OD)......20
Figure S27. ^1H NMR spectrum of **6** (600 MHz, CD_3OD) 21
Figure S28. ^{13}C and DEPT NMR spectra of **6** (150 MHz, CD_3OD)......21
Figure S29. 1H NMR spectrum of **7** (600 MHz, CD_3OD)......22
Figure S30. ^{13}C and DEPT NMR spectra of **7** (150 MHz, CD_3OD)......22
Figure S31. 1H NMR spectrum of **8** (600 MHz, CD_3OD)......23
Figure S32. ^{13}C and DEPT NMR spectra of **8** (150 MHz, CD_3OD)......23
Figure S33. HSQC spectrum of **8** 24
Figure S34. HMBC spectrum of **8** 24
Figure S35. 1H NMR spectrum of **9** (600 MHz, CD_3OD)......25
Figure S36. ^{13}C and DEPT NMR spectra of **9** (150 MHz, CD_3OD)......25
Figure S37. HSQC spectrum of **9** 26
Figure S38. HMBC spectrum of **9** 26

1. Spectroscopic data

Figure S1. 1H NMR spectrum of 1 (600 MHz, CD_3OD)

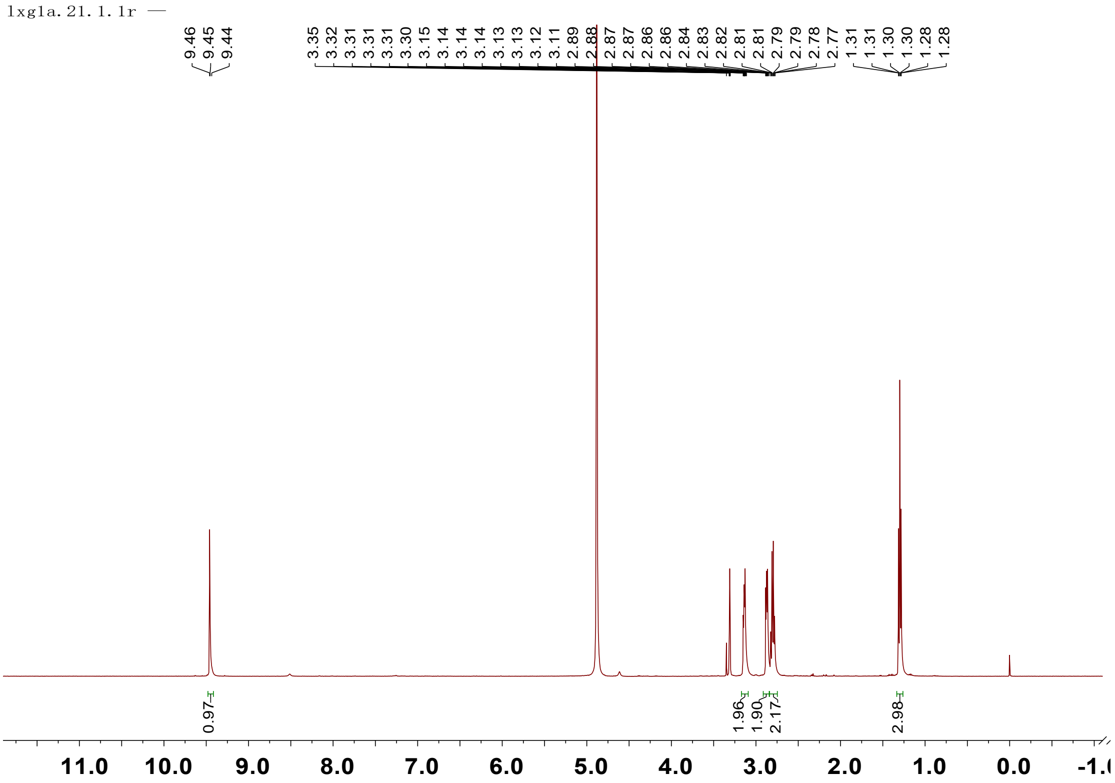

Figure S2. ^{13}C and DEPT NMR spectra of 1 (150 MHz, CD_3OD)

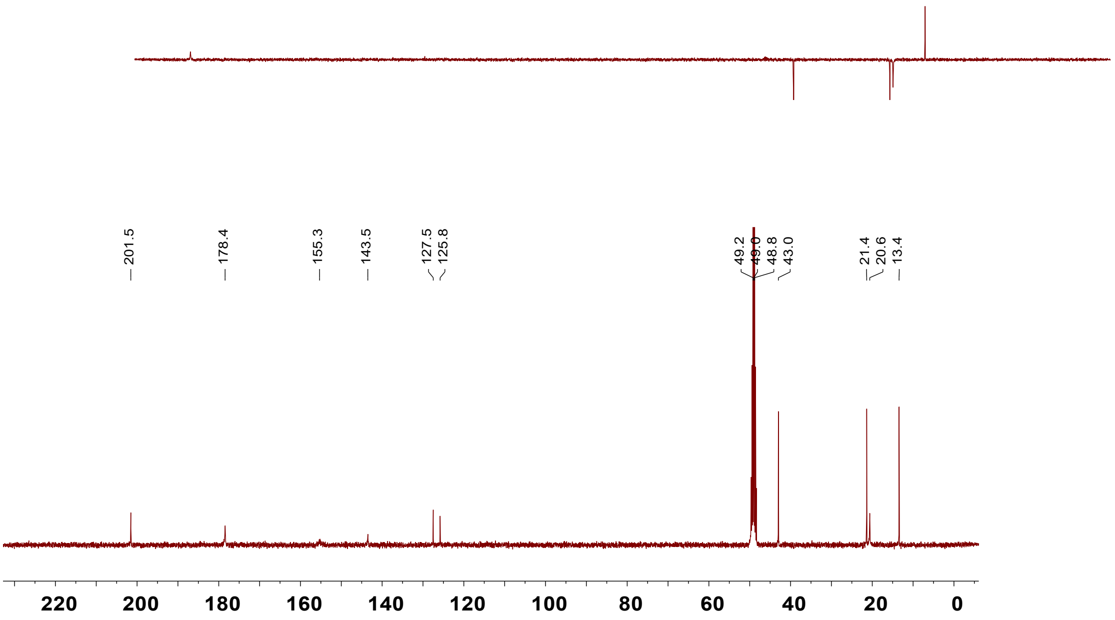

Figure S3. HMBC spectrum of 1

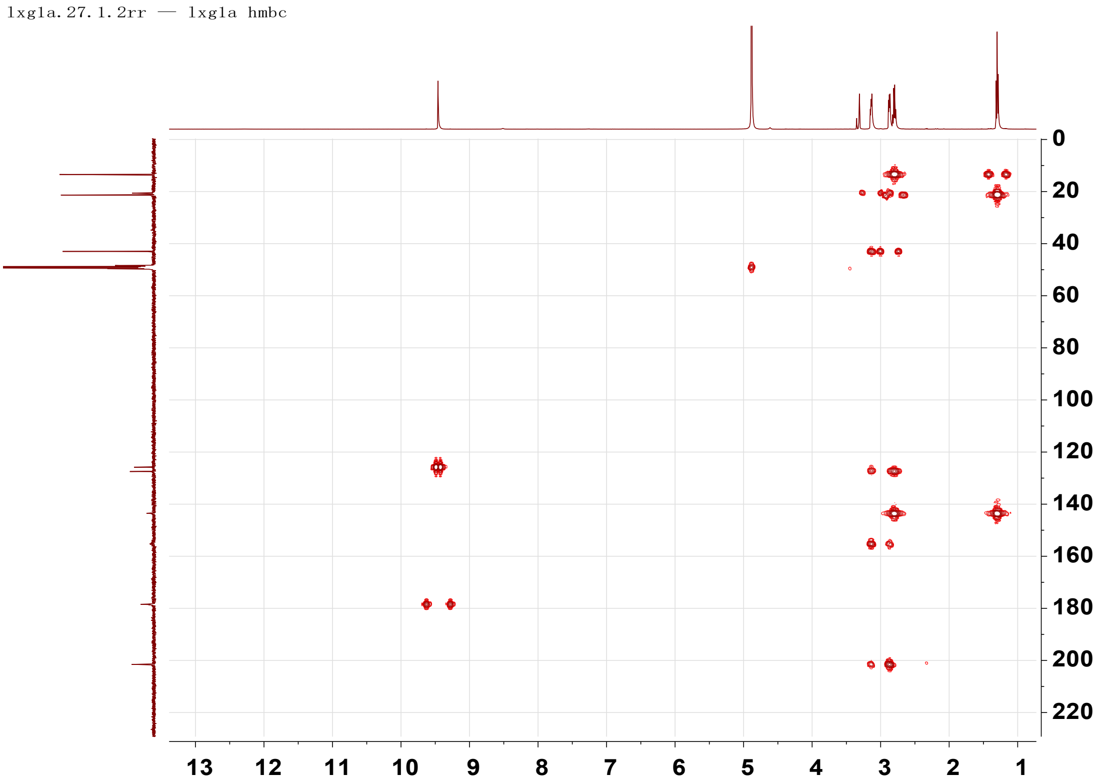

Figure S4. HRESIMS report of 1

Generic Display Report

Analysis Info

Analysis Name	D:\Data\GJH\ZZZ\1XG1A.d	Acquisition Date	3/7/2023 11:24:49 AM
Method	tune_pos_standard_20141031.m	Operator	Demo User
Sample Name	1XG1A	Instrument	maXis HD
Comment			

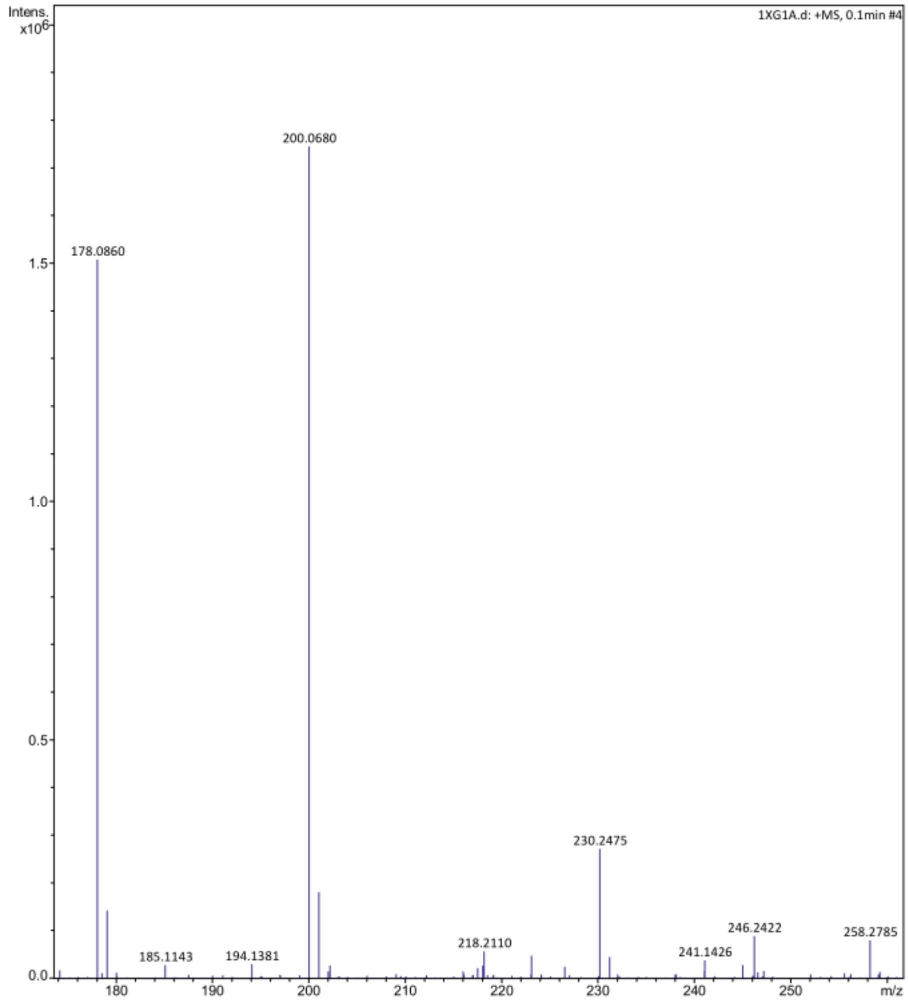

Bruker Compass DataAnalysis 4.4 printed: 3/31/2023 10:57:15 AM by: demo Page 1 of 1

Figure S5. IR report of 1

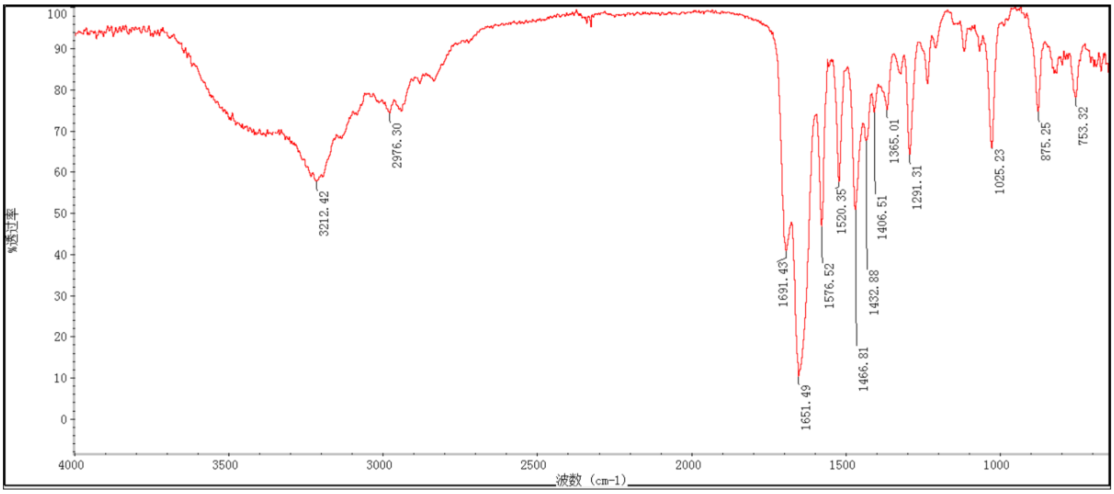

Figure S6. ^{1}H NMR spectrum of 2 (600 MHz, CD_3OD)

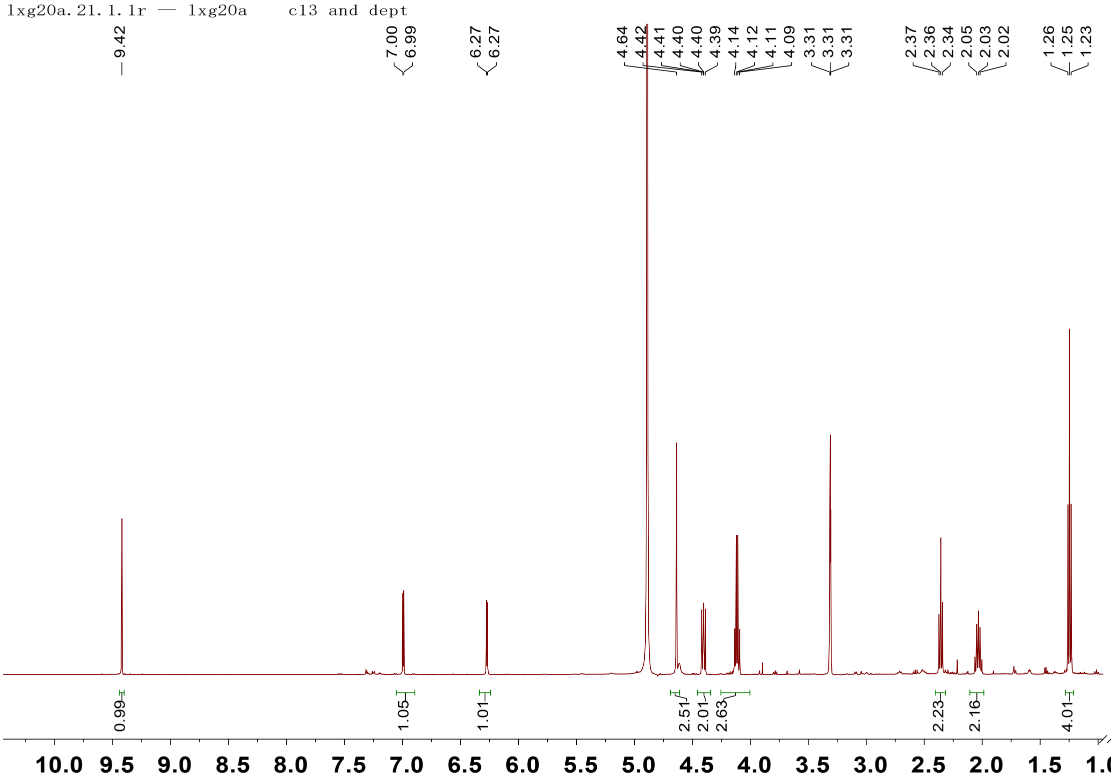

Figure S7. ^{13}C and DEPT NMR spectra of 2 (150 MHz, CD_3OD)

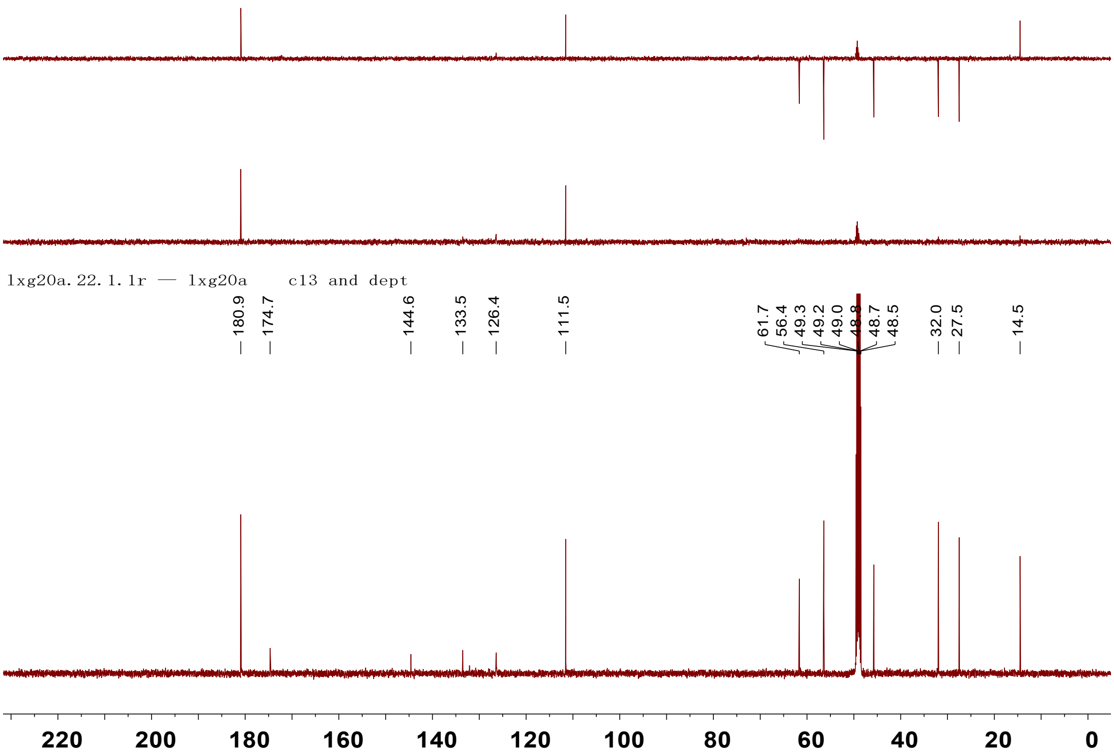

Figure S8. HSQC spectrum of 2

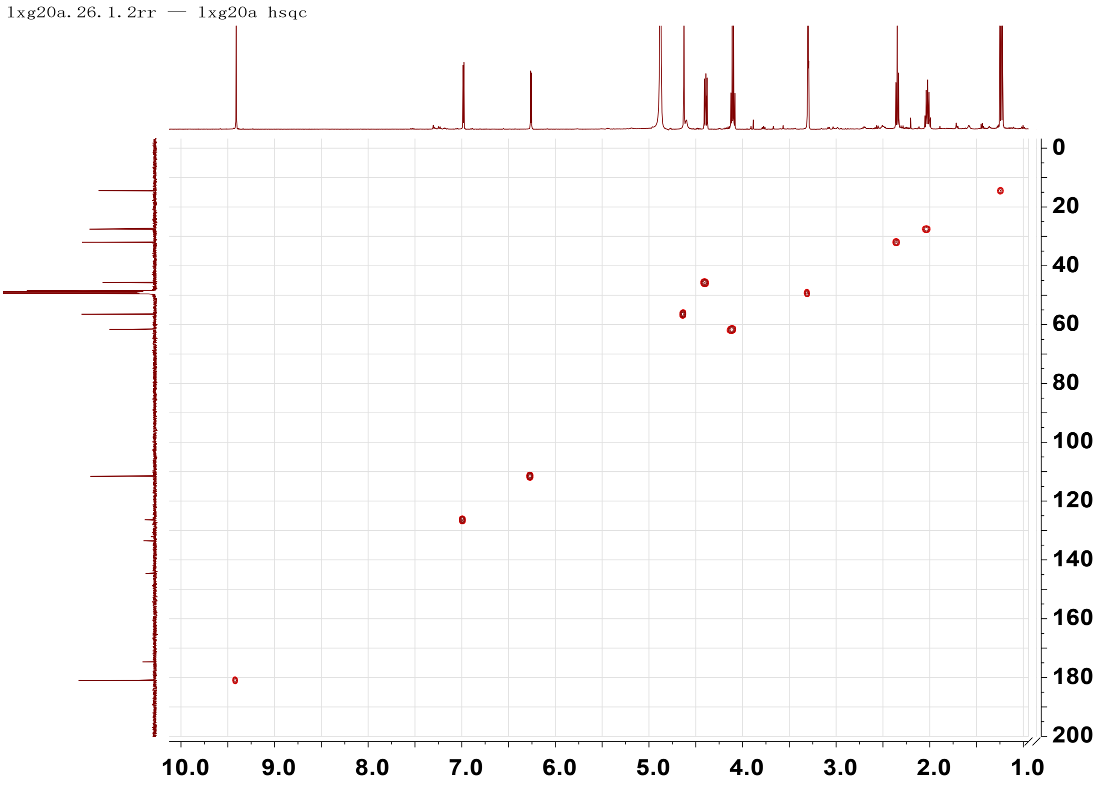

Figure S9. HMBC spectrum of 2

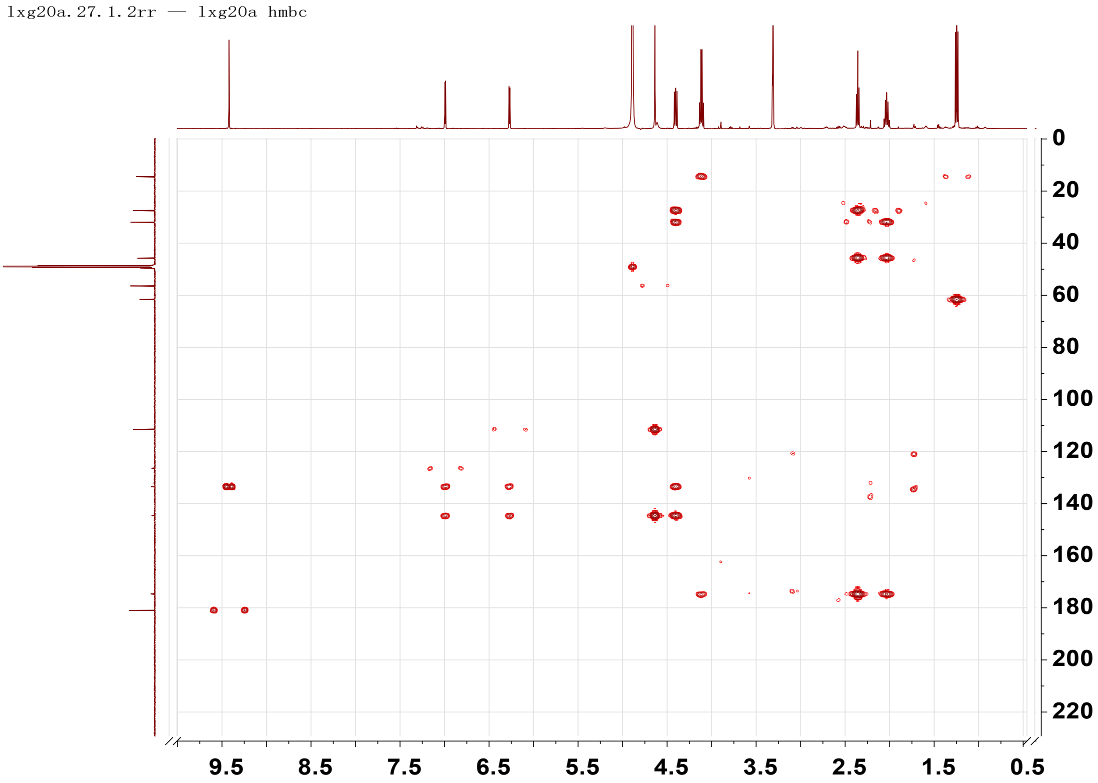

Figure S10. HRESIMS report of 2

Qualitative Analysis Report

**Data Filename**	lxg20a.d	**Sample Name**	lxg20a
**Sample Type**	Sample	**Position**	P1-A4
**Instrument Name**	Instrument 1	**User Name**	
**Acq Method**	s.m	**Acquired Time**	4/13/2018 2:13:55 PM
**IRM Calibration Status**	Success	**DA Method**	Default.m
**Comment**			

Sample Group **Info.**
Acquisition SW 6200 series TOF/6500 series
Version Q-TOF B.05.01 (B5125.2)

User Spectra

Fragmentor Voltage	Collision Energy	Ionization Mode
135	0	ESI

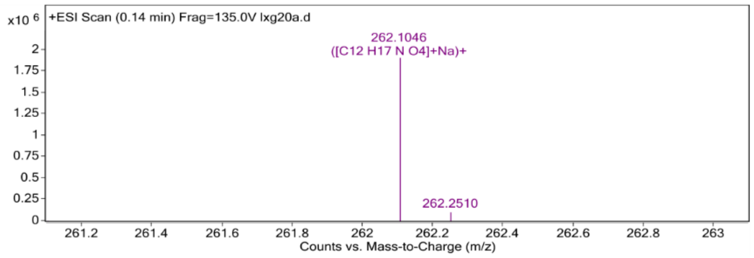

Peak List

m/z	z	Abund	Formula	Ion
102.1269	1	513321.69		
200.1268	1	393223.44		
222.111	1	1169930.88		
262.1046	1	1912241.25	C12 H17 N O4	(M+Na)+
278.0785	1	541678.94		
461.2267	1	366489.53		
501.2225	1	1421629.38		
502.2253	1	391769.81		

Formula Calculator Element Limits

Element	Min	Max
C	3	60
H	0	120
O	0	30
N	0	3

Formula Calculator Results

Formula	CalculatedMass	CalculatedMz	Mz	Diff. (mDa)	Diff. (ppm)	DBE
C12 H17 N O4	239.1158	262.1050	262.1046	0.40	1.53	5.0000

--- End Of Report ---

Figure S11. IR report of 2

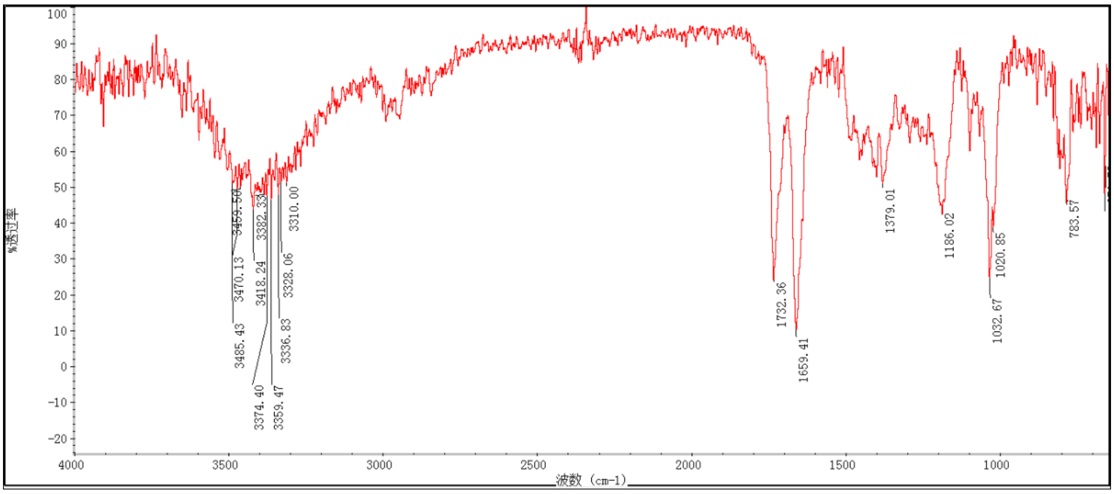

Figure S12. ^{1}H NMR spectrum of 3 (600 MHz, CD_3OD)

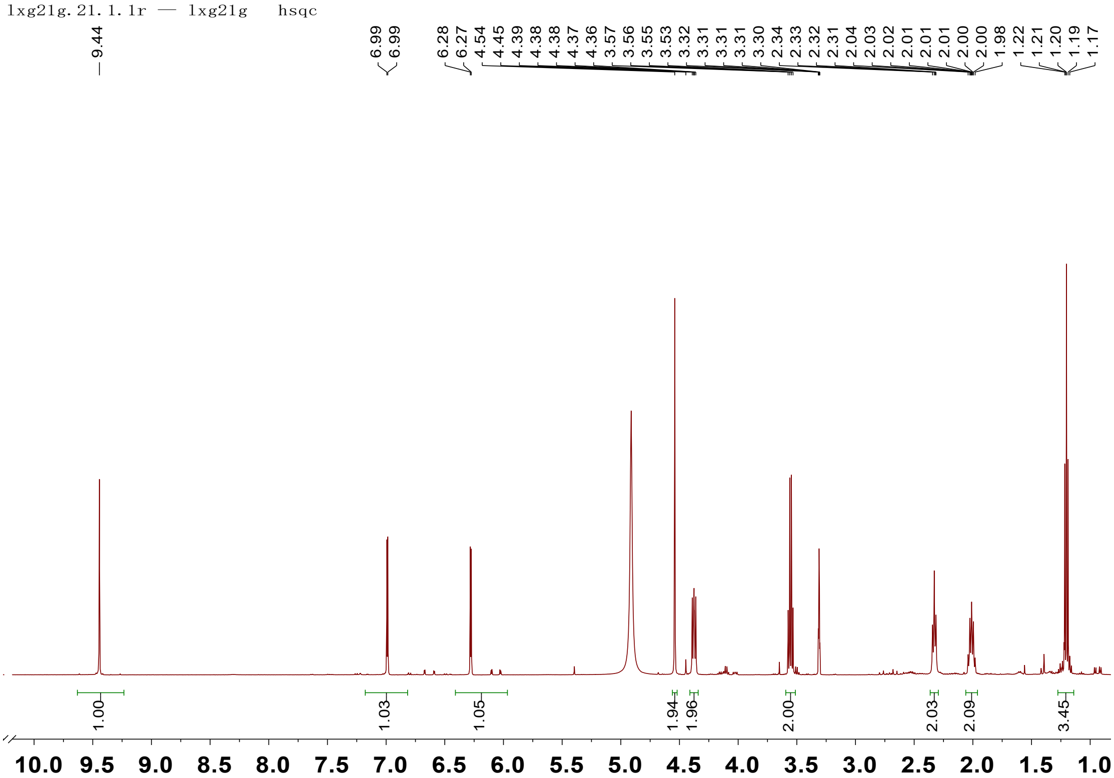

Figure S13. ^{13}C and DEPT NMR spectra of 3 (150 MHz, CD_3OD)

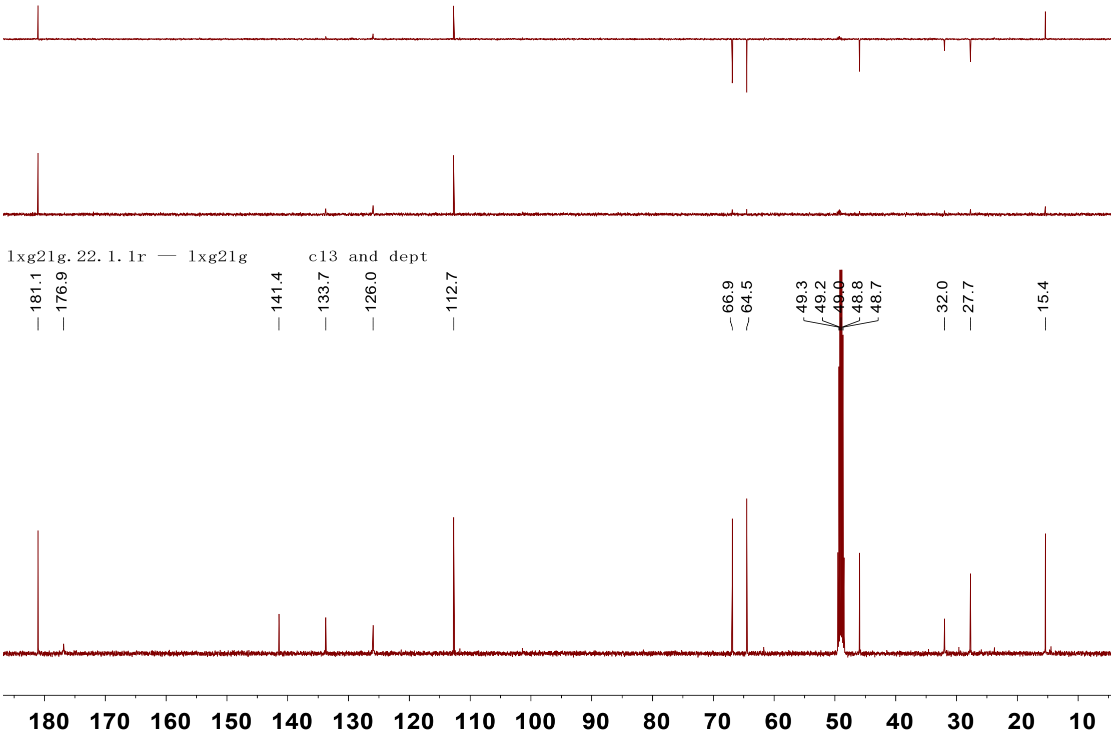

Figure S14. HSQC spectrum of 3

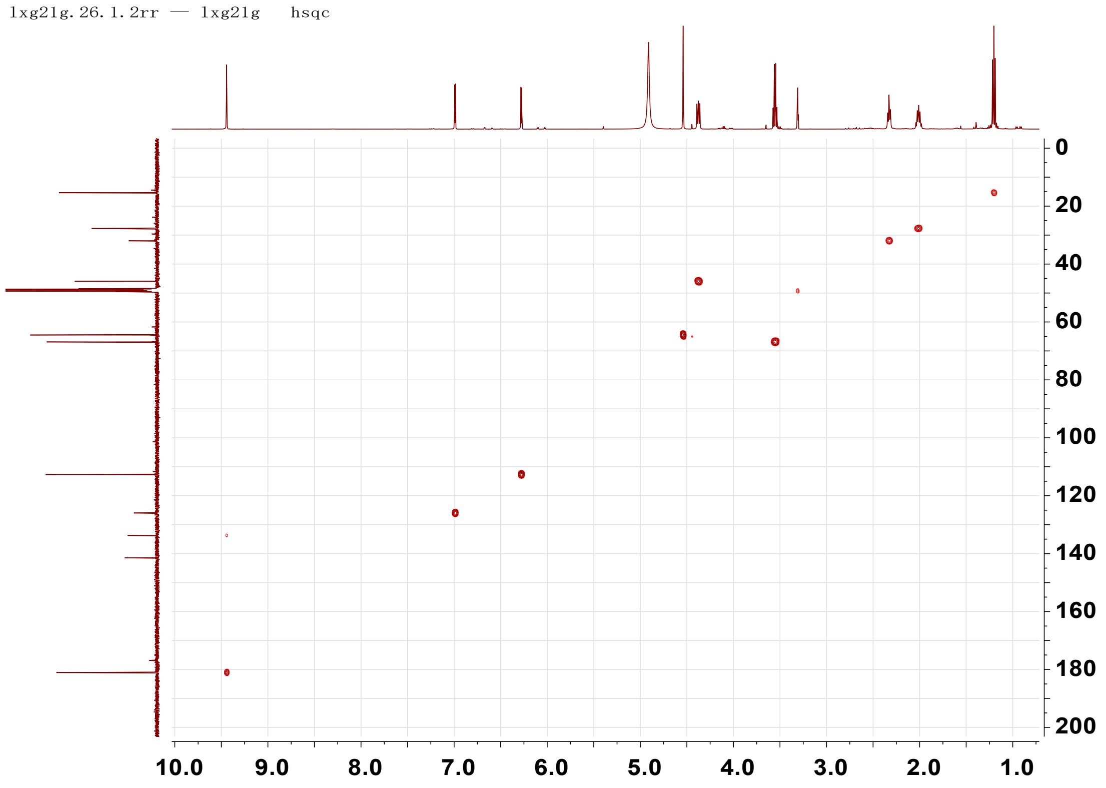

Figure S15. HMBC spectrum of 3

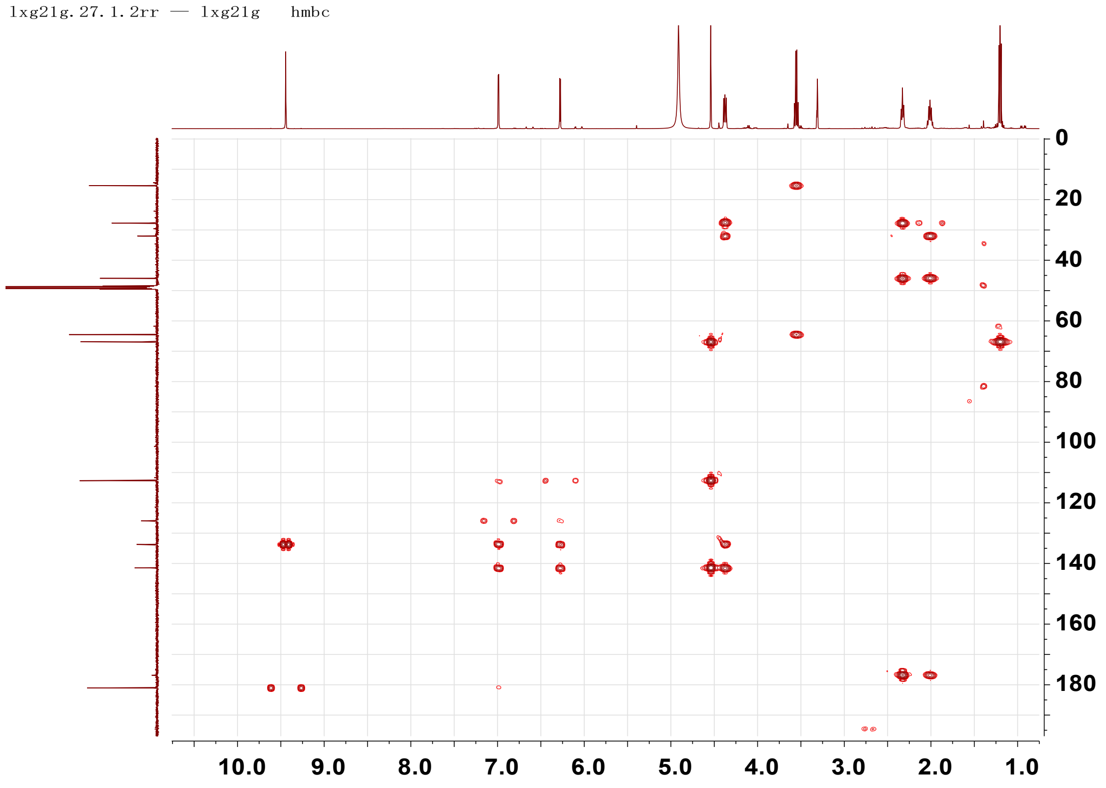

Figure S16. HRESIMS report of 3

Qualitative Analysis Report

**Data Filename**	lxg21g.d	**Sample Name**	lxg21g
**Sample Type**	Sample	**Position**	P1-A5
**Instrument Name**	Instrument 1	**User Name**	
**Acq Method**	s.m	**Acquired Time**	5/3/2018 10:10:32 AM
**IRM Calibration Status**	Success	**DA Method**	Default.m
**Comment**			

Sample Group **Info.**
Acquisition SW 6200 series TOF/6500 series
Version Q-TOF B.05.01 (B5125.2)

User Spectra

Fragmentor Voltage	Collision Energy	Ionization Mode
100	0	ESI

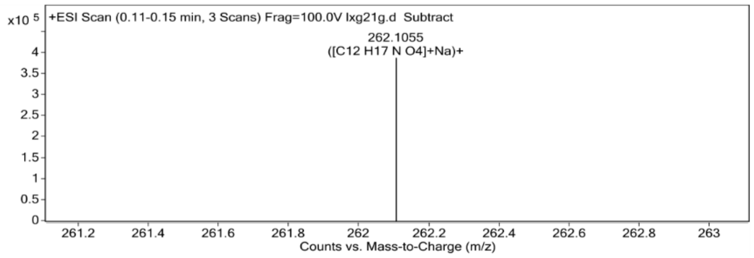

Peak List

m/z	z	Abund	Formula	Ion
194.081	1	35750.83		
262.1055	1	389479.88	C12 H17 N O4	(M+Na)+
263.1085	1	49929	C12 H17 N O4	(M+Na)+
278.0791	1	127673.75		
501.2211	1	110860.15		
502.2235	1	29897.03		
523.2029	1	27776.94		
540.2571	1	29902.53		

Formula Calculator Element Limits

Element	Min	Max
C	3	60
H	0	120
O	0	30
N	0	3

Formula Calculator Results

Formula	CalculatedMass	CalculatedMz	Mz	Diff. (mDa)	Diff. (ppm)	DBE
C12 H17 N O4	239.1158	262.1050	262.1055	-0.50	-1.91	5.0000

--- End Of Report ---

Figure S17. IR report of 3

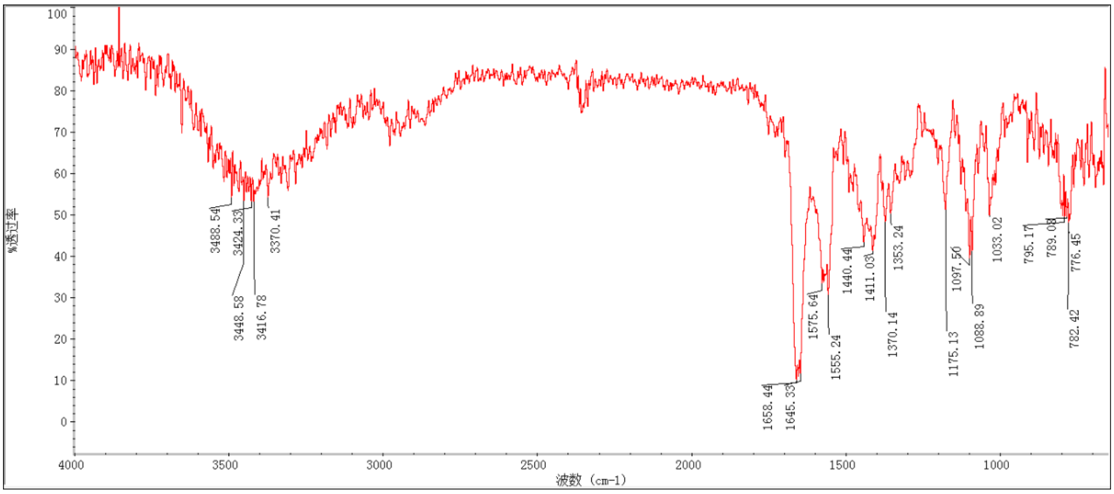

Figure S18. ^{1}H NMR spectrum of 4 (600 MHz, CD_3OD)

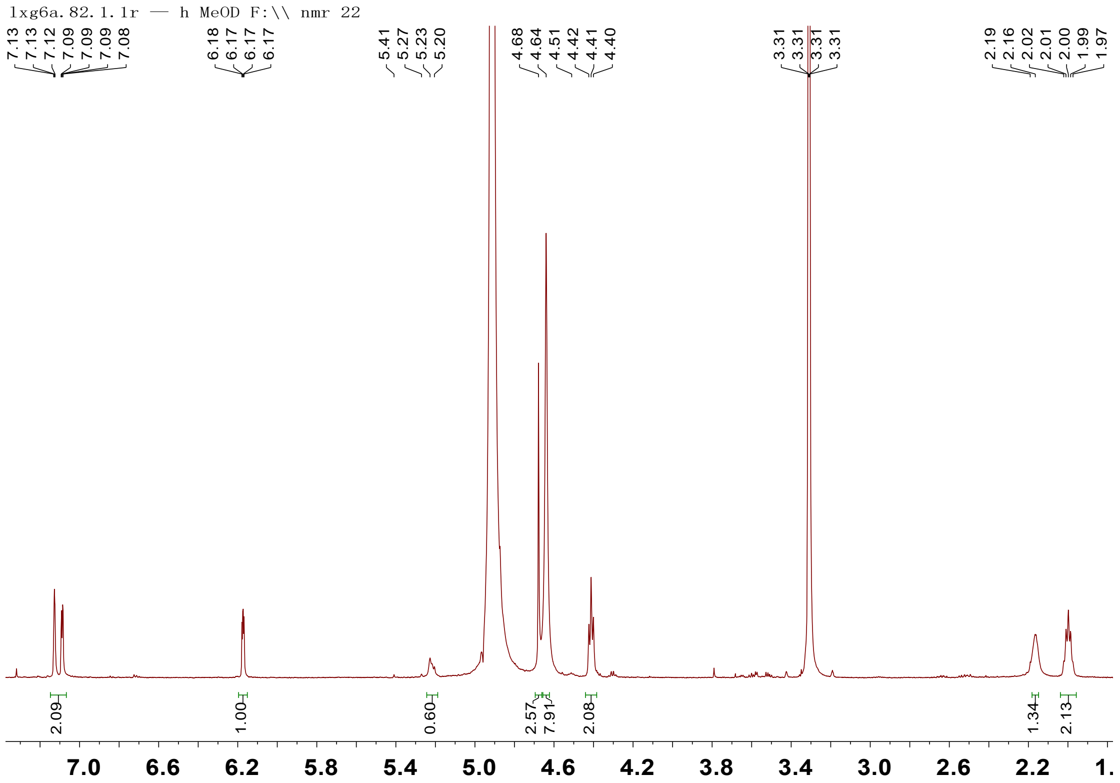

Figure S19. ^{13}C and DEPT NMR spectra of 4 (150 MHz, CD_3OD)

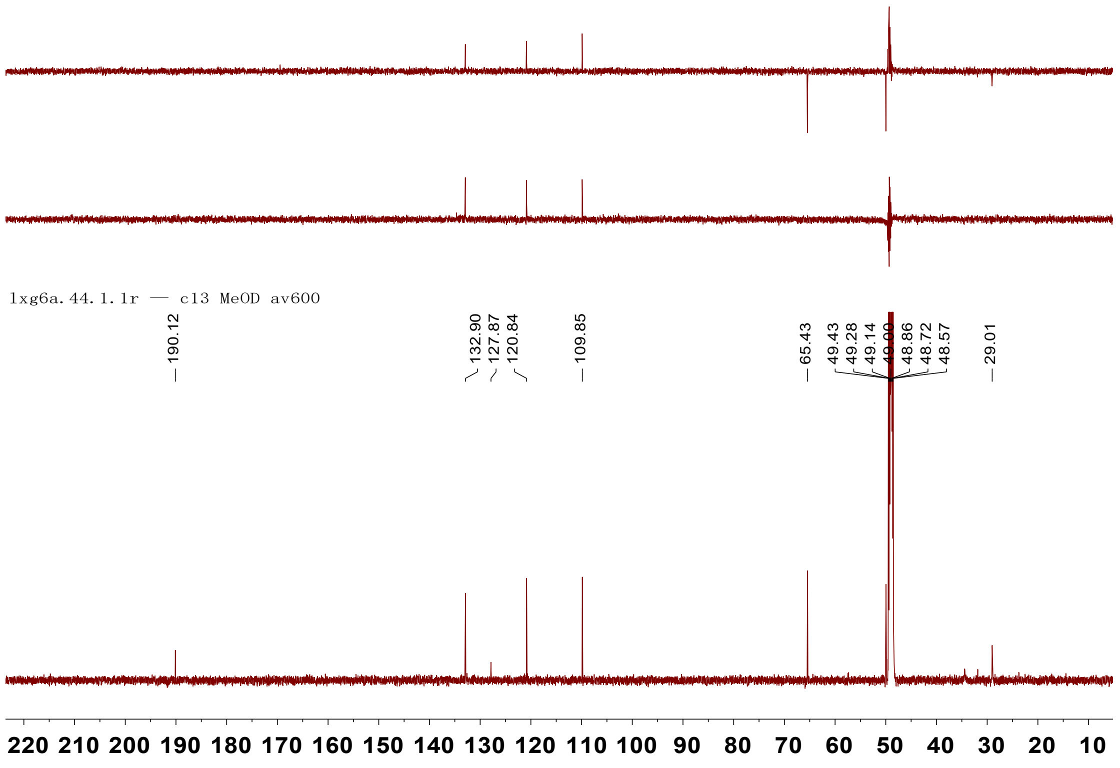

Figure S20. HSQC spectrum of 4

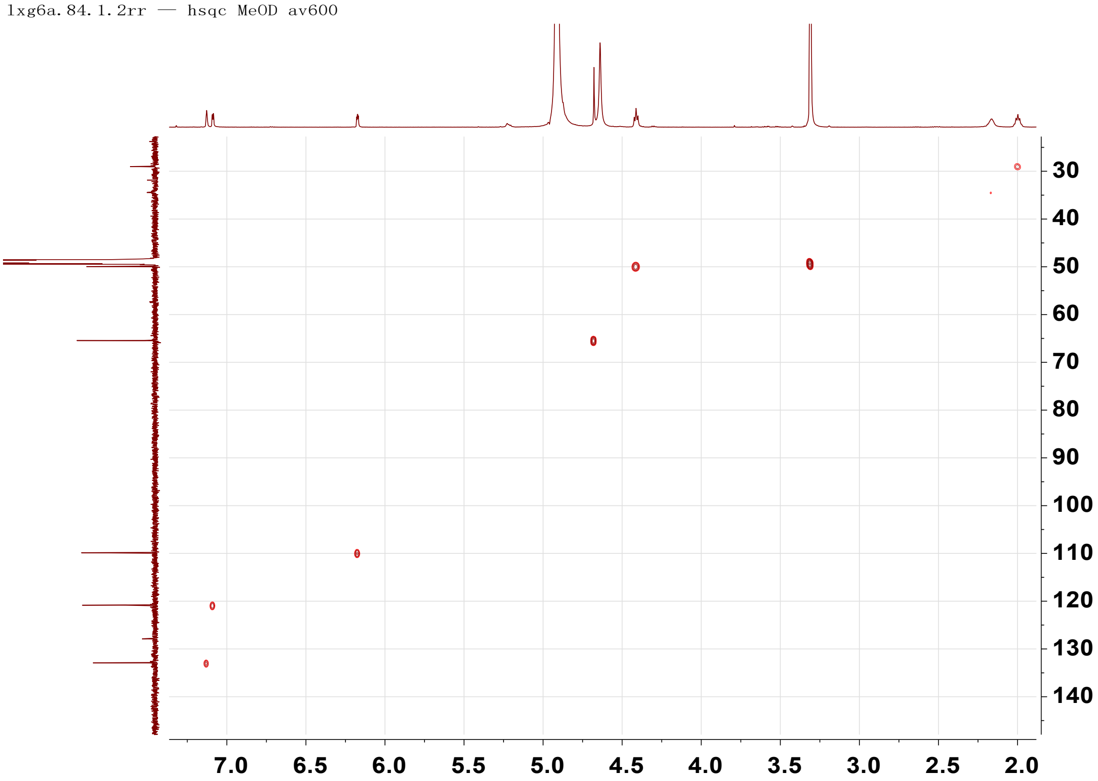

Figure S21. ^{1}H-^{1}H COSY spectrum of 4

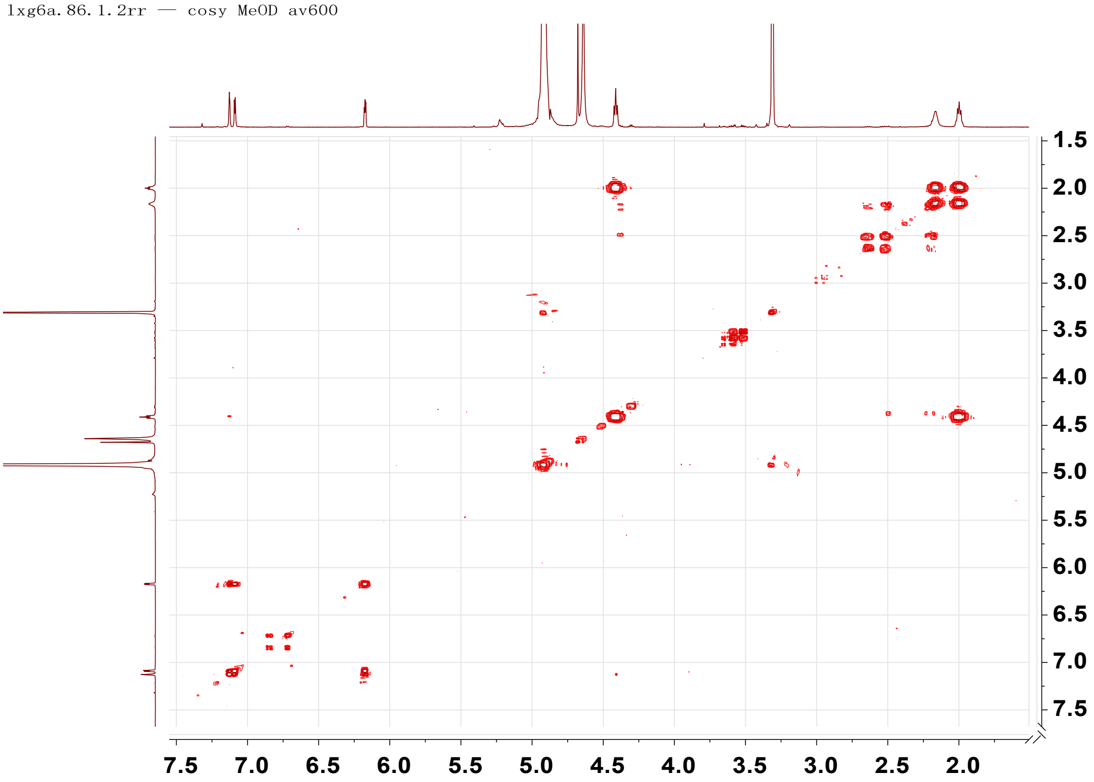

Figure S22. HMBC spectrum of 4

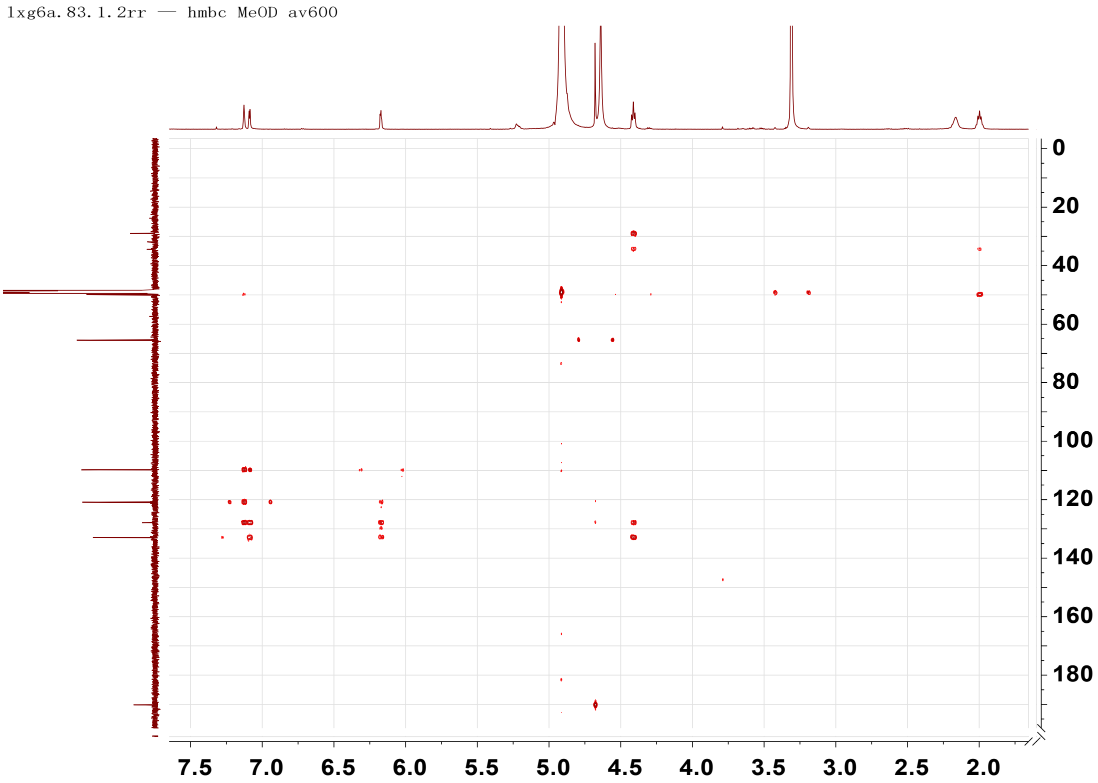

Figure S23. HRESIMS report of 4

Data File: E:\DATA\2018\0128\lxg6a.lcd

Elmt	Val.	Min	Max	Elmt	Val.	Min	Max	Elmt	Val.	Min	Max	Elmt	Val.	Min	Max	Use Adduct
H	1	0	100	O	2	0	50	Si	4	0	0	Br	1	0	0	Na
C	4	0	100	F	1	0	0	S	2	0	0	I	3	0	0	
N	3	0	10	Na	1	0	0	Cl	1	0	0					

Error Margin (ppm):	5	DBE Range:	-2.0 - 100.0	Electron Ions:	both
HC Ratio:	unlimited	Apply N Rule:	yes	Use MSn Info:	yes
Max Isotopes:	all	Isotope RI (%):	1.00	Isotope Res:	10000
MSn Iso RI (%):	75.00	MSn Logic Mode:	AND	Max Results:	10

Event#: 1 MS(E+) Ret. Time : 0.507 Scan# : 77

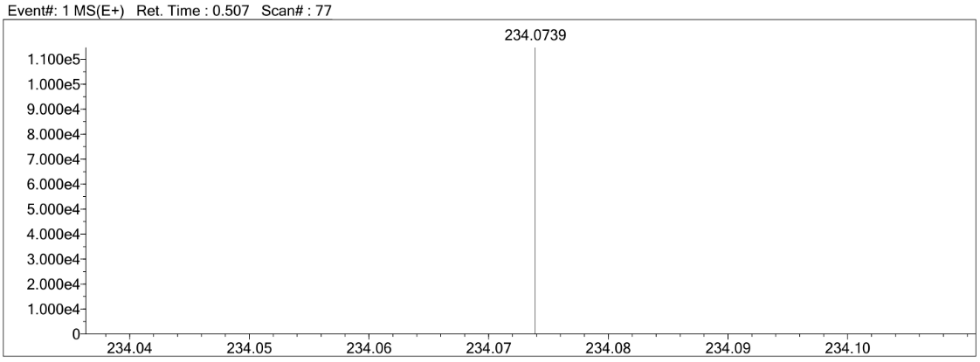

Measured region for 234.0739 m/z

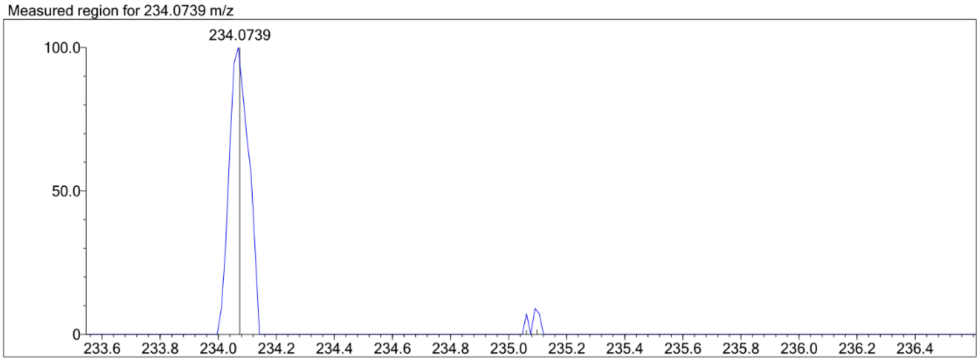

C10 H13 N O4 [M+Na]+ : Predicted region for 234.0737 m/z

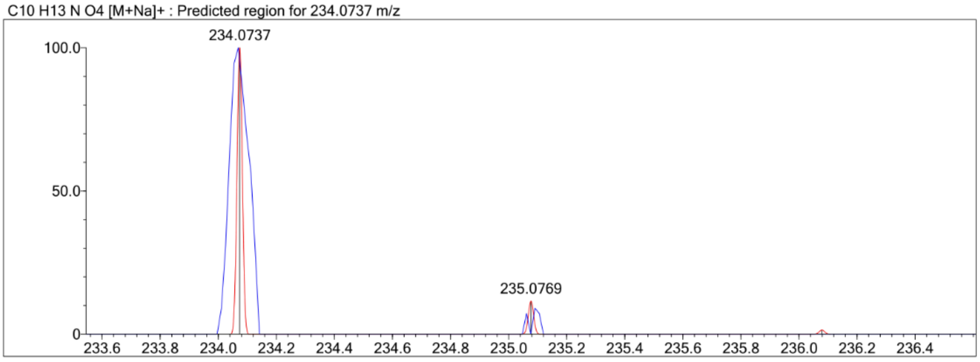

Formula (M)	Ion	Meas. m/z	Pred. m/z	Df. (mDa)	Df. (ppm)	DBE
C10 H13 N O4	[M+Na]+	234.0739	234.0737	0.2	0.85	5.0

Figure S24. IR report of 4

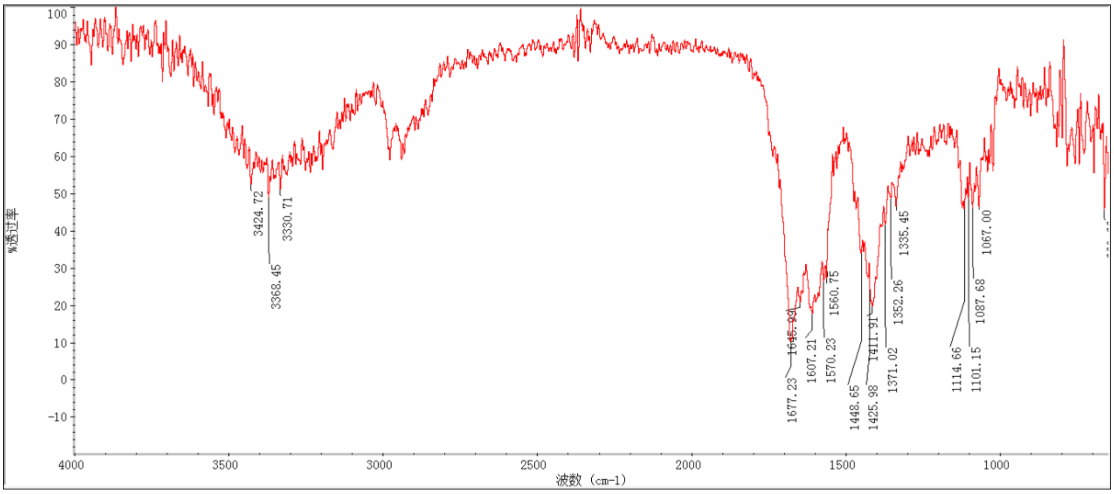

Figure S25. ^{1}**H NMR spectrum of 5 (600 MHz,** CD_3OD**)**

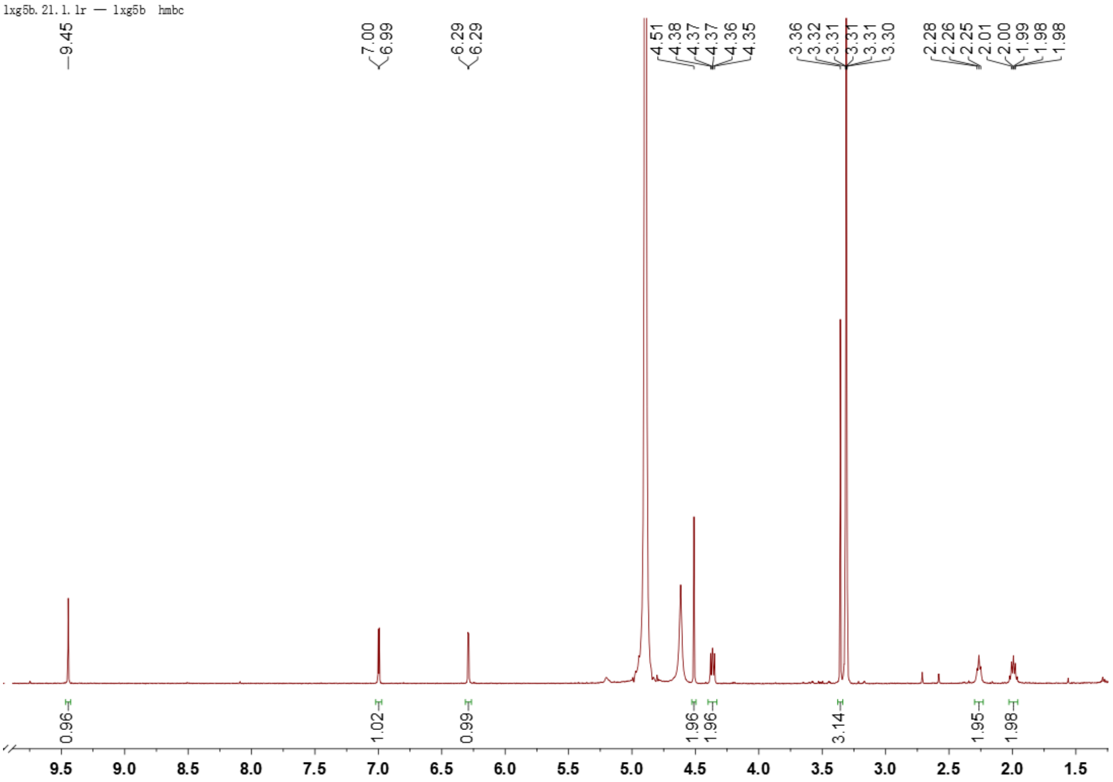

Figure S26. ^{13}**C and DEPT NMR spectra of 5 (150 MHz,** CD_3OD**)**

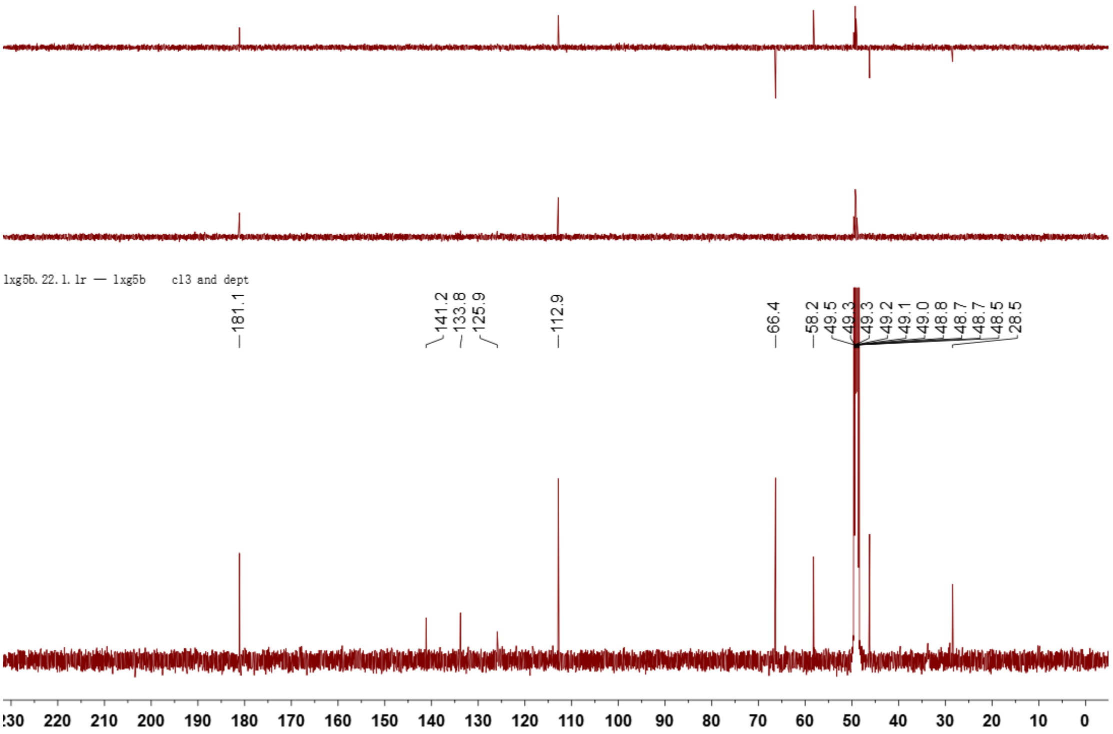

Figure S27. ^{1}H **NMR spectrum of 6 (600 MHz, CD_3OD)**

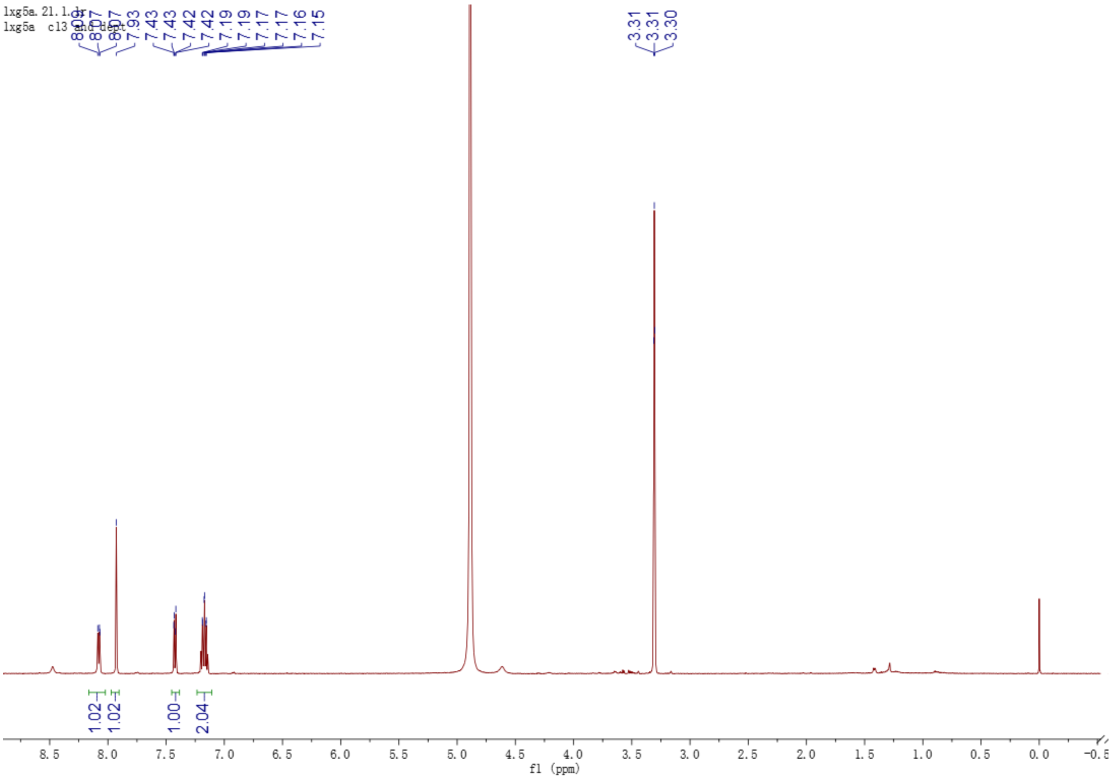

Figure S28. ^{13}C **and DEPT NMR spectra of 6 (150 MHz, CD_3OD)**

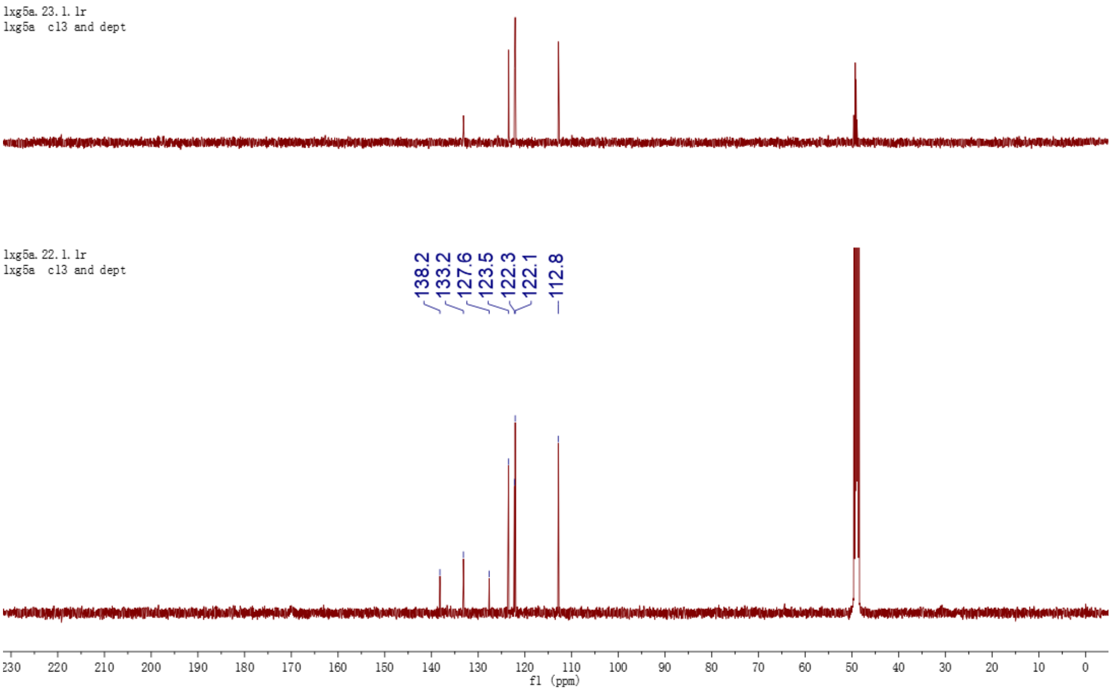

Figure S29. 1H NMR spectrum of 7 (600 MHz, CD_3OD)

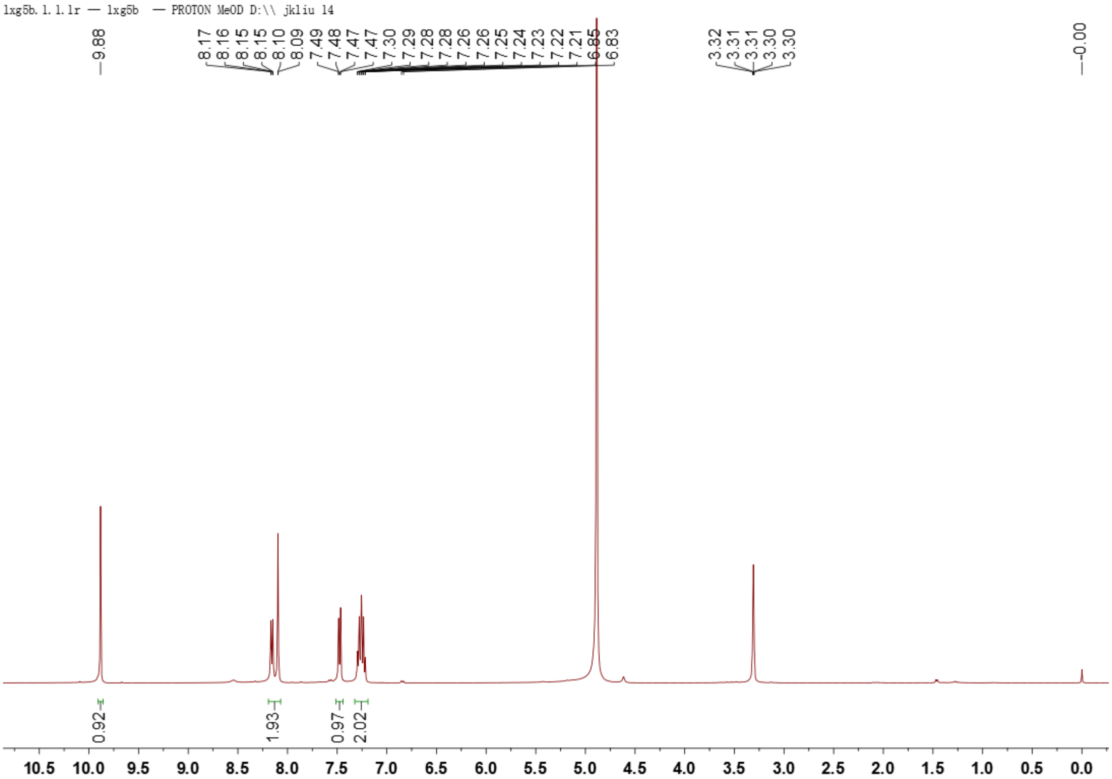

Figure S30. ^{13}C and DEPT NMR spectra of 7 (150 MHz, CD_3OD)

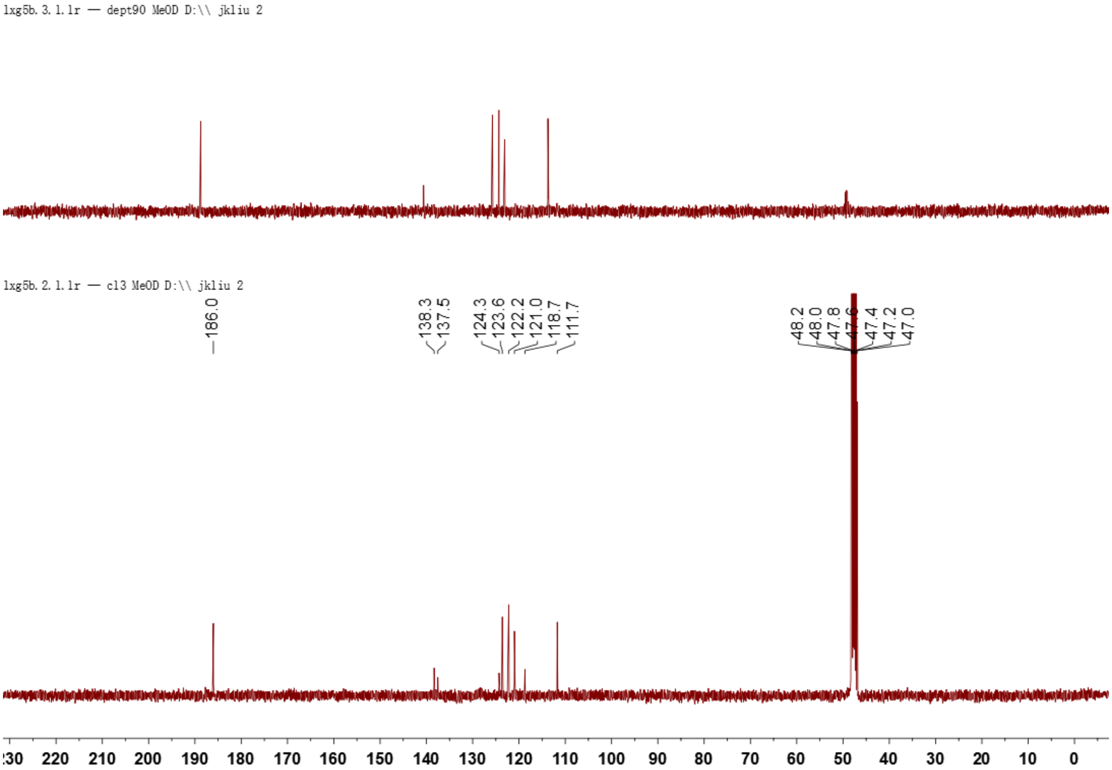

Figure S31. 1H NMR spectrum of 8 (600 MHz, CD_3OD)

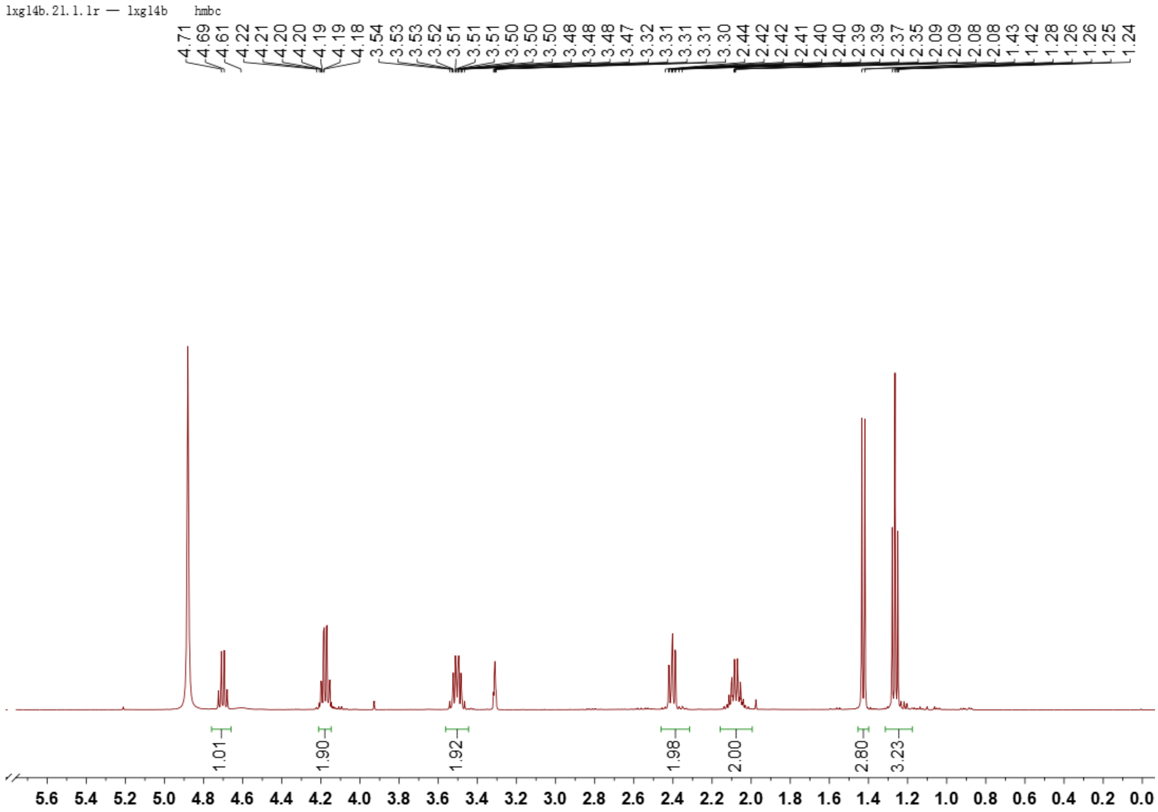

Figure S32. ^{13}C and DEPT NMR spectra of 8 (150 MHz, CD_3OD)

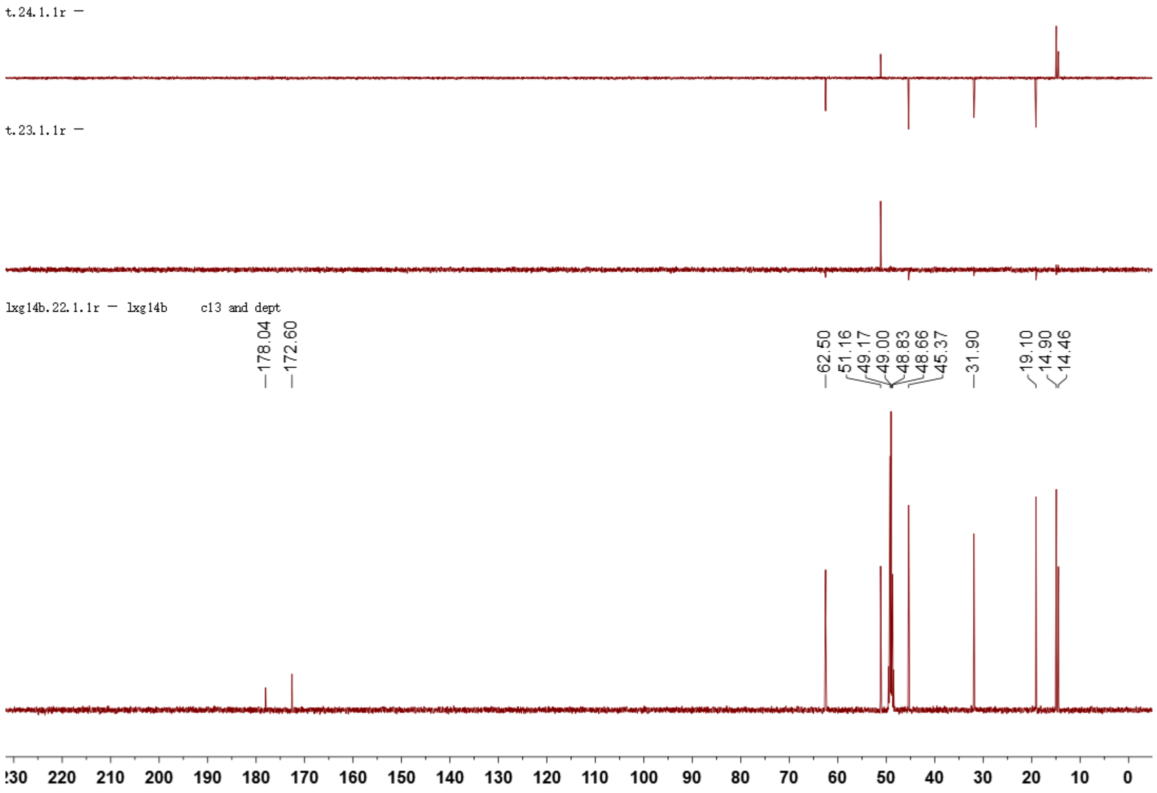

Figure S33. HSQC spectrum of 8

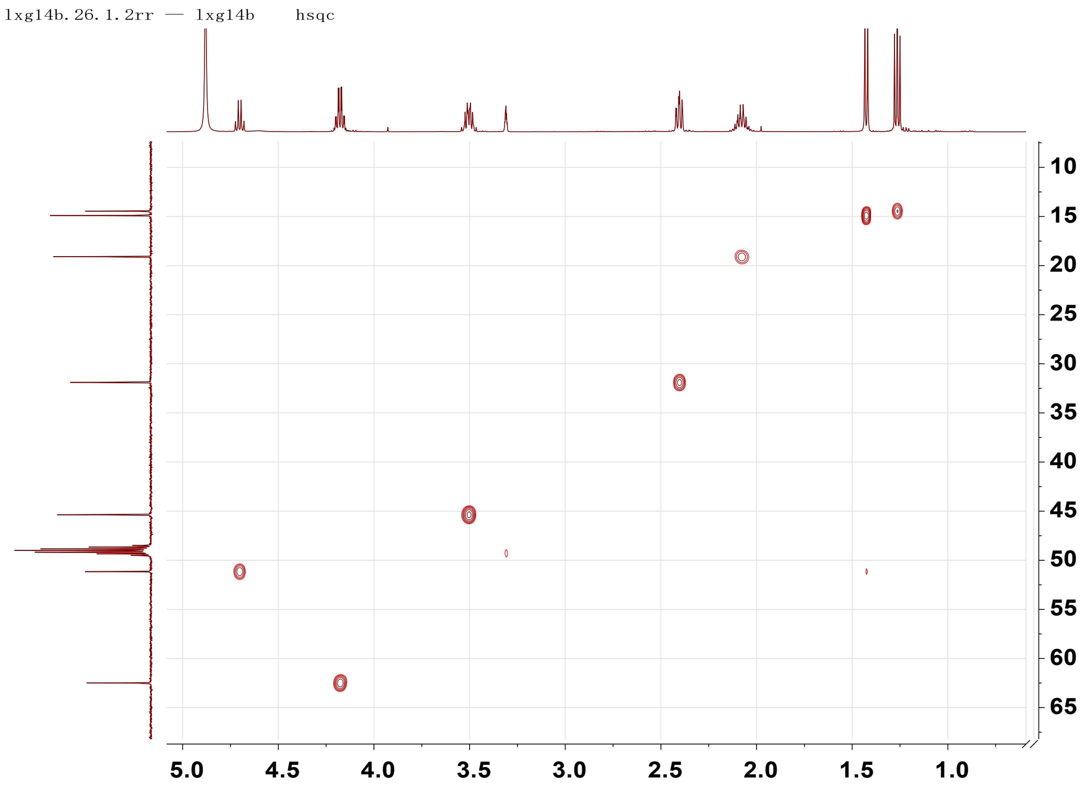

Figure S34. HMBC spectrum of 8

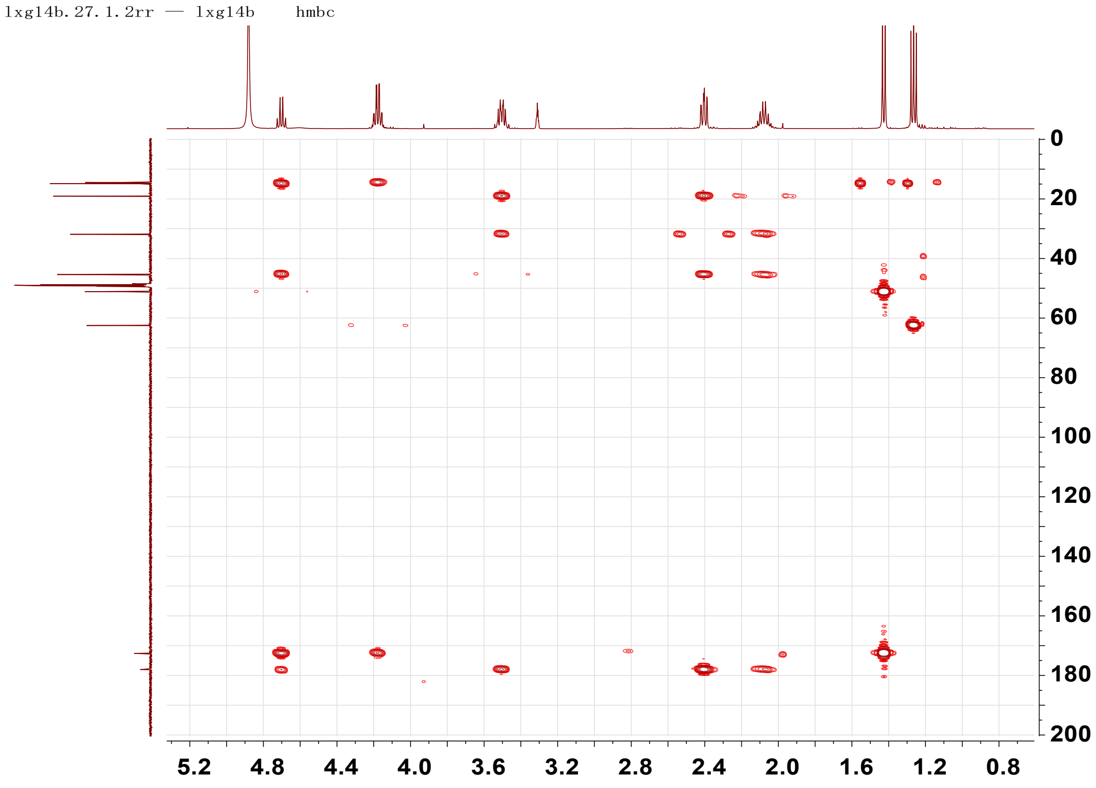

Figure S35. 1H NMR spectrum of 9 (600 MHz, CD_3OD)

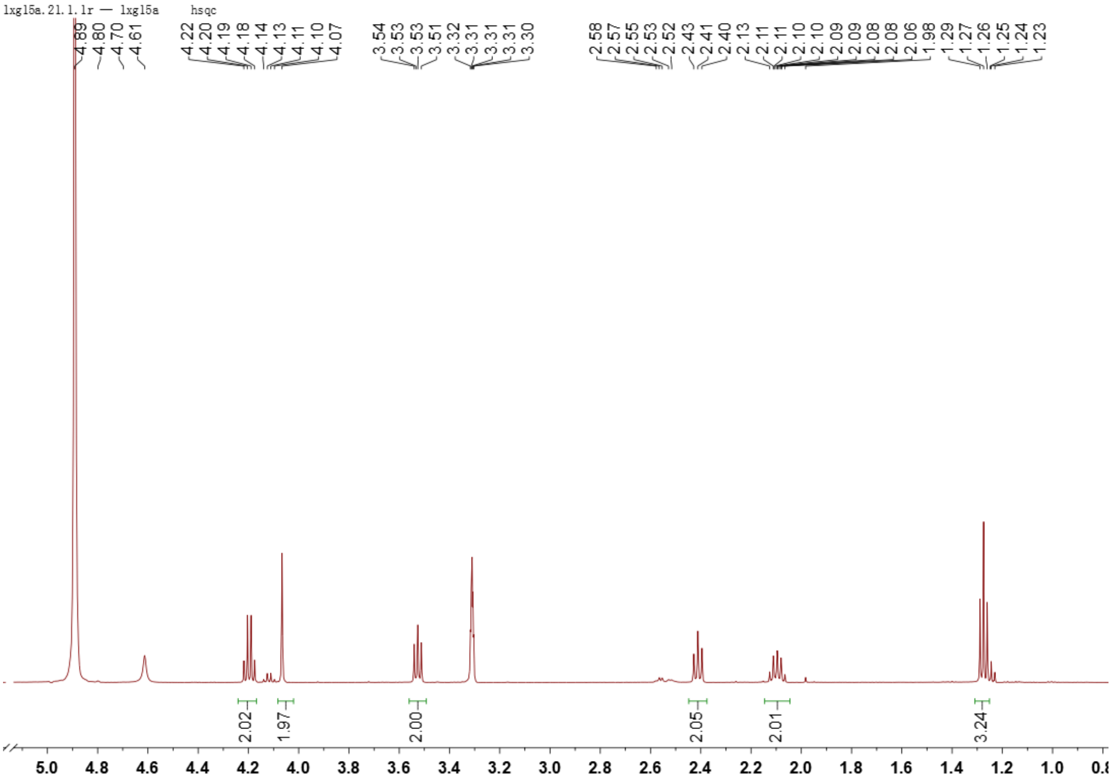

Figure S36. ^{13}C and DEPT NMR spectra of 9 (150 MHz, CD_3OD)

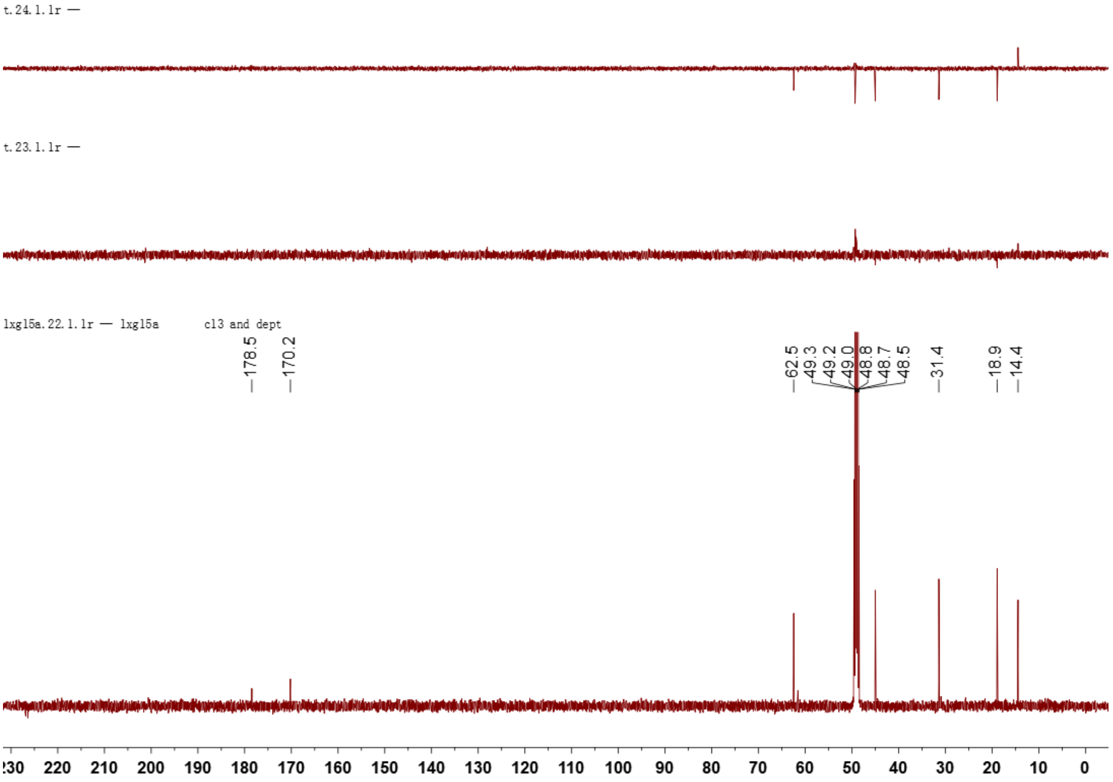

Figure S37. HSQC spectrum of 9

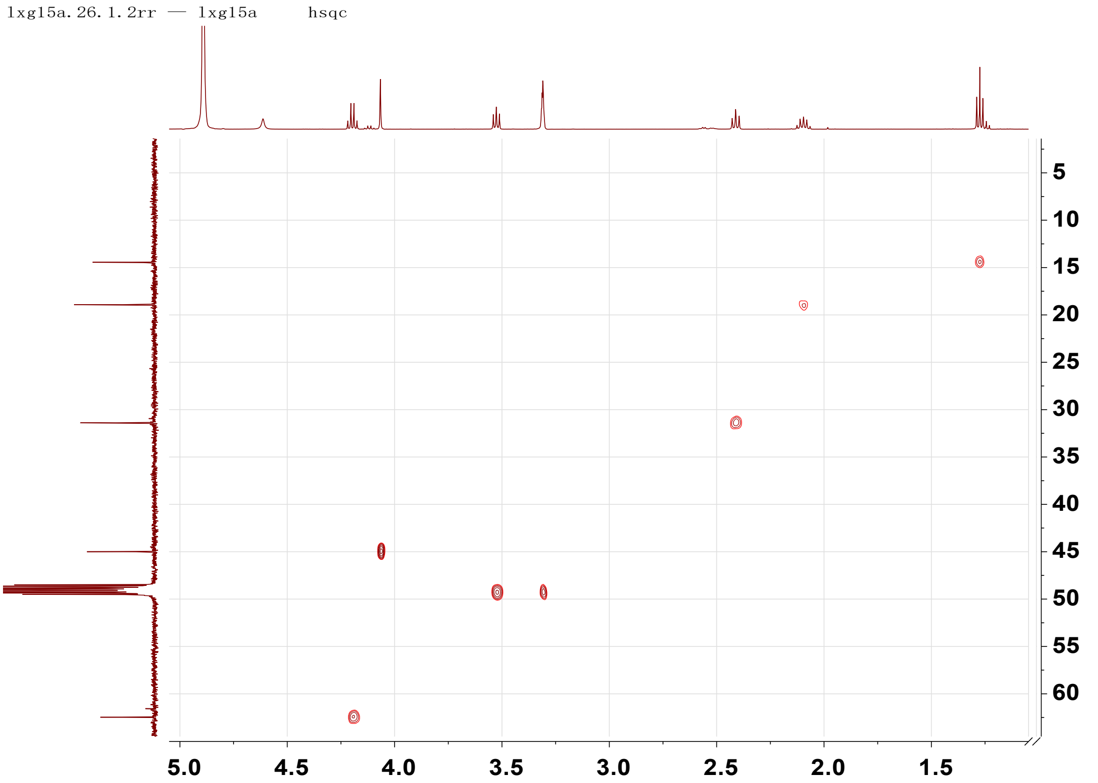

Figure S38. HMBC spectrum of 9

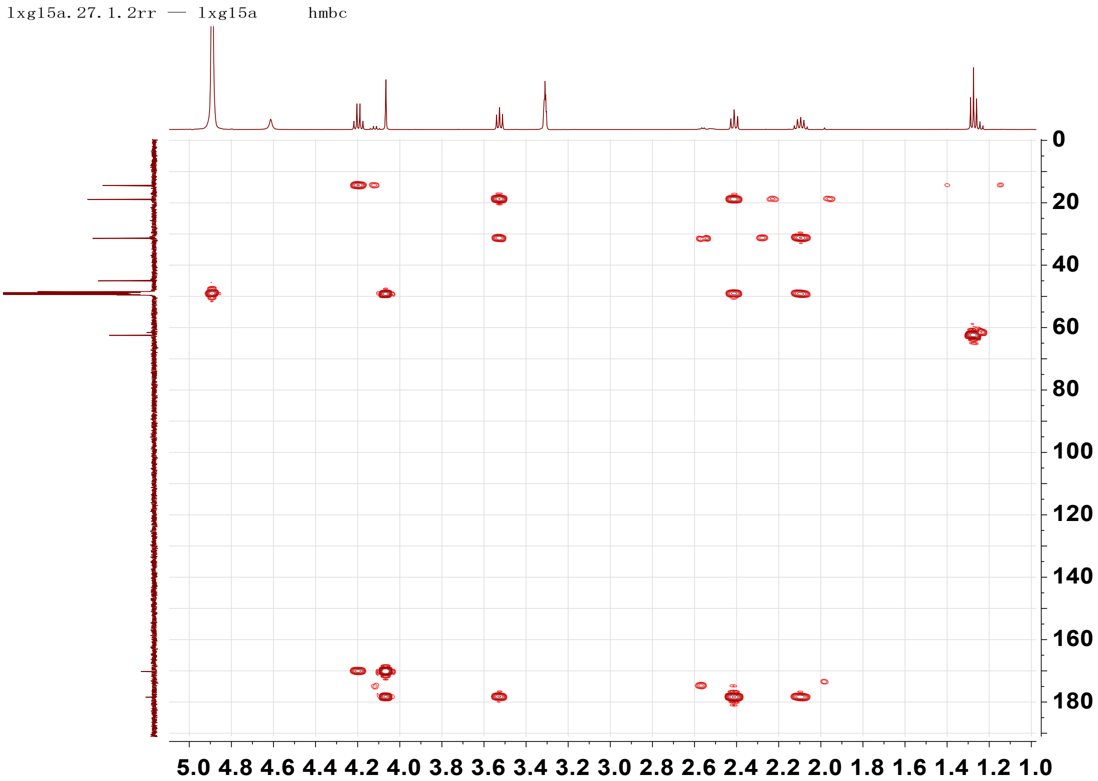

Supplement: RA-013-D3RA02672H-s001 [file RA-013-D3RA02672H-s001.pdf]
